# Growth suppression by dual BRAF(V600E) and NRAS(Q61) oncogene expression is mediated by SPRY4 in melanoma

**DOI:** 10.1038/s41388-018-0632-2

**Published:** 2019-01-16

**Authors:** Raj Kumar, Ching-Ni Njauw, Bobby Y. Reddy, Zhenyu Ji, Anpuchchelvi Rajadurai, Nikolai Klebanov, Hensin Tsao

**Affiliations:** 1 Department of Dermatology and Wellman Center for Photomedicine, Massachusetts General Hospital, Harvard Medical School, Boston, MA USA; 2 Department of Dermatology, Massachusetts General Hospital, Harvard Medical School, Edwards 211 50 Blossom Street, Boston, MA USA

**Keywords:** Melanoma, Senescence

## Abstract

The underlying forces that shape mutational patterns within any type of cancer have been poorly characterized. One of the best preserved exclusionary relationships is that between BRAF(V600E) and NRAS(Q61) in melanomas. To explore possible mechanisms which could explain this phenomenon, we overexpressed *NRAS*(Q61) in a set of *BRAF*(V600E) melanoma lines and vice versa. Controlled expression of a second activating oncogene led to growth arrest (“synthetic suppression”) in a subset of cells, which was accompanied by cell cycle arrest and senescence in several melanoma cell lines along with apoptosis. Through differential gene expression analysis, we identified *SPRY4* as the potential mediator of this synthetic response to dual oncogene suppression. Ectopic introduction of *SPRY4* recapitulated the growth arrest phenotype of dual BRAF(V600E)/NRAS(Q61) expression while *SPRY4* depletion led to a partial rescue from oncogenic antagonism. This study thus defined SPRY4 as a potential mediator of synthetic suppression, which is likely to contribute to the observed exclusivity between BRAF(V600E) and NRAS(Q61R) mutations in melanoma. Further leverage of the SPRY4 pathway may also hold therapeutic promise for *NRAS*(Q61) melanomas.

## Introduction

Within tumors, mutational patterns reflect strong evolutionary forces and yet represent only a single snapshot of a complex physiology. Over 45,000 tumor specimens have been subjected to various analyses including whole exome sequencing (www.cbioportal.org). One of the most commonly activated networks is the RAS-MAPK, which impacts both cancer cell proliferation and survival [1, 2]. While recurrent oncogenic lesions in BRAF (pV600) and N/K/HRAS (pG12/13, p.Q61) predominate within the RAS-MAPK pathway, they are rarely identified in conjunction within any single tumor specimen [3, 4].

Among the myriad of tumors analyzed to date, cutaneous melanoma bears some of the highest mutational burdens [5]. Thus, it is somewhat surprising that exclusion between *BRAF* c.1799T>A(V600E) and *NRAS* c.181C>A (Q61K)/*NRAS* c.182A>G (Q61R) mutations is so pronounced in melanoma; there is only a single melanoma tumor specimen out of 366 sequenced which harbored concurrent *BRAF* c.1799T>A(V600E)/*BRAF* c.1798G>A (V600M) and *NRAS* c.37G>C (G13R) mutations (TCGA-ES-A2NC sample; www.bioportal.org). The biological pressures that govern the emergence and patterning of these activating alleles have not been well characterized. A priori, redundancy and antagonism, through growth arrest, apoptosis, senescence or other means, are both possible explanations. Under a redundancy model, the second oncogenic hit would have minimal functional impact and thus exist as a low probability “passenger” oncogene. Alternatively, under an antagonistic framework, an additional activating allele would functionally interfere with tumor growth and thus drop out of the final tumor population. Petti et al. showed that forced expression of NRAS(Q61R) in a single BRAF(V600E) melanoma line led to growth arrest and induction of SA-ß-gal [6], consistent with senescence. These results suggest that the introduction of a rival oncogene impinges on two cancer processes: oncogene-induced senescence (OIS) and synthetic lethality. In the former, expression of a strong activating allele in the context of a noncancerous cell leads to the onset of senescence due to a battery of compensatory mechanisms [7] such as normal telomerase activity. Since melanoma cells have already breached OIS during their initial transformation, it would be more appropriate to describe oncogene exclusion as “secondary OIS”. For synthetic lethality, the viability of a cancer cell is compromised when two mutations co-exist whether these changes be activating or loss-of-function [8]. While synthetic lethal interactions may be condition-dependent, there is much enthusiasm about identifying such genetic pairs since the potency of synthetically lethal interactions could offer clues about potentially “druggable” targets. Furthermore, since dual mutant states may be antagonistic but not necessarily lethal, perhaps “synthetic suppression” could be a more encompassing term. Along these lines, we set out to more deeply characterize the mechanism(s) which proscribe the concurrence of BRAF(pV600E) and NRAS(pQ61) mutations in melanoma with an eye towards novel pathways which could countermand constitutive BRAF or NRAS signaling.

## Results

### Oncogene exclusion and synthetic suppression

**Fig. 1:**
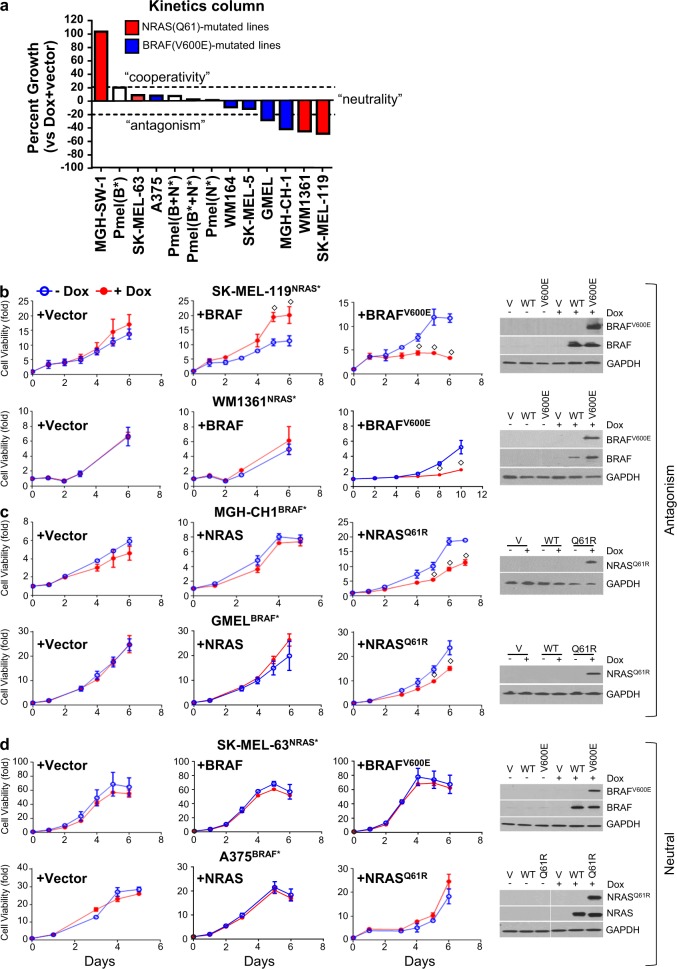
Differential cell growth response upon rival oncogene expression. The rival oncogene was induced with doxycycline (50–100 ng/ml) and subjected for 6 days cell viability assays using cell-titer-glow reagent. **a** A panel of four isogeneic stable NRAS(Q61)/Tet-On BRAF(V600E) and five BRAF(V600E)/Tet-On-NRAS(Q61R) mutant cell lines and an immortalized primary human melanocyte line (Pmel) were assayed for cell viability at fifth day following rival oncogene induction with doxycycline. Cell lines showing antagonism such as **b** two NRAS(Q61R)/Tet-On-BRAF(V600E) and **c** two BRAF(V600E)/Tet-On-NRAS(Q61R). Cell lines showing cooperativity/neutral such as **d** one BRAF(V600E)/Tet-On-NRAS(Q61R) and one NRAS(Q61R)/Tet-On-BRAF(V600E). The protein expression was confirmed by western blotting. For each cell line, cell viability was performed independently more than three times in triplicates. Student’s *t* test, doxycycline vs. no-doxycycline, ^◇^*p* *≤* 0.05

We first set out to establish the broader context of oncogene exclusion by examining the impact of dual oncogenes in native NRAS(Q61) and BRAF(V600E) lines. To avoid unwarranted negative selection during the introduction of the “rival” oncogene (i.e. NRAS(Q61) for BRAF(V600E) melanoma lines and BRAF(V600E) for NRAS(Q61) melanoma lines), we used a Tet-On system to synchronize expression of the second allele in a panel of four isogeneic stable NRAS(Q61R/K) + doxycycline-induced Tet-On- BRAF(V600E) lines (designated as “NRAS* + iBRAF*”) and five BRAF(V600E) + doxycycline-induced Tet-On-NRAS(Q61R) lines (designated as “BRAF* + iNRAS*”) (Fig. 1a) along with an immortalized primary human melanocyte line (Pmel). The rival oncogene was induced with doxycycline (50–100 ng/ml) and subjected for 6-day cell viability assays. Using an arbitrary definition of ±20% above vector for “cooperativity” and “antagonism”, one of the four (red bars) NRAS* + iBRAF* lines exhibited significant cooperativity in growth (MGH-SW-1^NRAS*^: +102.5%) while the other two demonstrated significant antagonism (SK-MEL-119^NRAS*^: −49.4% and WM1361^NRAS*^: −45.8%). Among (blue bars) BRAF*+iNRAS* lines, interactions were neutral except for GMEL^BRAF*^ and MGH-CH-1^BRAF*^, which exhibited growth decrements of −29.1 and −42.6%, respectively, with the induction of the exogenous NRAS* mutation. In the Pmel line (an immortalized melanocyte line with wild-type *BRAF* and wild-type *NRAS*), we observed better growth with iBRAF(V600E) and combined iBRAF(WT) + iNRAS(Q61R) than with either iBRAF(V600E) + iNRAS(Q61R) or iNRAS(Q61R) alone; all the lines, however, increased <20%. Morphological changes and fluorescent protein expression were validated by fluorescence microscopy and protein expression was confirmed by western blotting at sixth day of cell viability (Fig. 1, Figs. S2, S3). These results indicate that coexpression of BRAF* and NRAS* can, at least in a subset of lines, lead to growth arrest and perhaps contribute to the clinical observation of oncogene exclusion.

**Fig. 2:**
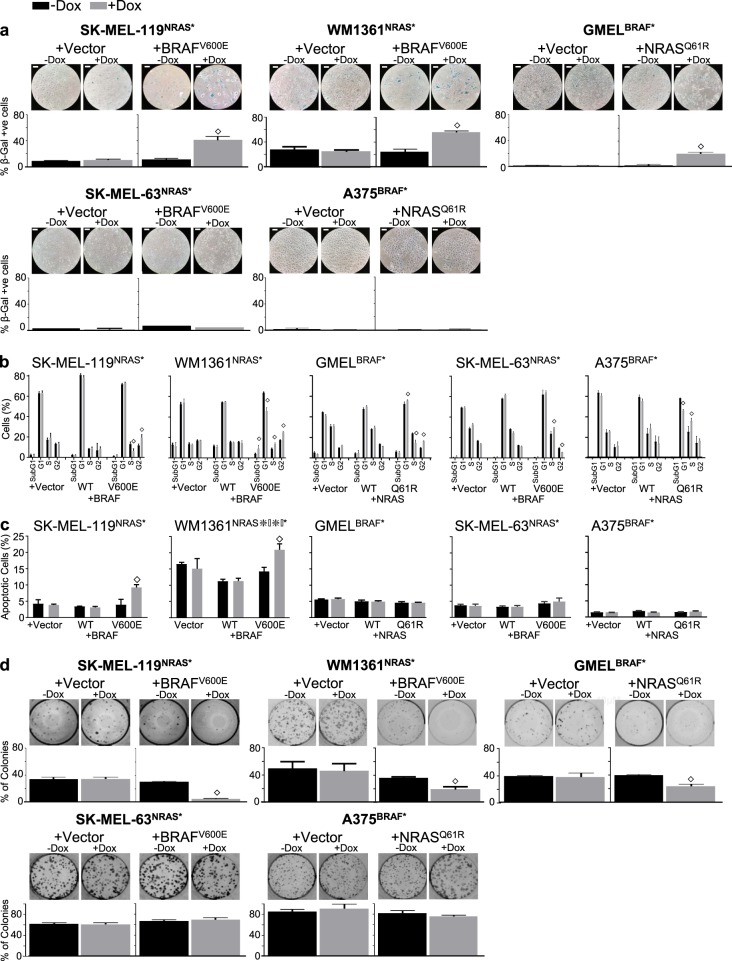
Ectopic induction rival oncogene inhibit melanoma cell growth and enhance associated phenotypes. On fifth day following rival oncogene induction with doxycycline (50–100 ng/ml), three antagonistic NRAS* + iBRAF* lines (SK-MEL-119^NRAS*^ and WM1361^NRAS*^, GMEL^BRAF*^) and two neutral BRAF* + iNRAS* (SK-MEL-63^NRAS*^ and A375^BRAF*^) lines were assayed for their phenotypic dependence. **a** Senescence was detected by senescence-associated expression of β-galactosidase (SA-β-gal) staining, (**b**) different phases of cell cycle were detected with PI staining and **c** apoptosis was detected by Annexin-V staining and **d** colony formation was detected by 0.1% of crystal violet in 24 well plates. Student’s *t* test, doxycycline vs. no-doxycycline, ^◇^*p* *≤* 0.05. Bar, 40 µm. The data presented are representative of three independent experiments

To isolate the effects of the mutation from the general increases in BRAF or NRAS protein levels, the two most suppressed NRAS(Q61R) lines (SK-MEL-119^NRAS*^ and WM1361^NRAS*^), two most suppressed BRAF(V600E) cell lines (MGH-CH-1^BRAF*^ and GMEL^BRAF*^) and two “neutral” BRAF(V600E) and NRAS(Q61K) cell lines (A375^BRAF*^ and SK- MEL-63^NRAS*^) were selected for further analysis. As shown in Fig. 1b, ectopic *BRAF(V600E)* expression in SK-MEL-119^NRAS*^ and WM1361^NRAS*^ both demonstrated significant growth suppression. Interestingly, forced expression of wild-type *BRAF*, especially in SK-MEL-119^NRAS*^, enhanced growth, which is consistent with NRAS(Q61R)’s upstream disposition. Similarly, *NRAS(Q61R)* induction in MGH-CH-1^BRAF*^ and GMEL^BRAF*^ (Fig. 1c) both confirmed significant growth suppression though ectopic wild-type *NRAS* expression did not appear to alter growth kinetics significantly in these BRAF(V600E) cells. As expected, the *iBRAF*(V600E) and *iNRAS*(Q61R) alleles had no effect on the SK-MEL-63^NRAS*^ and A375^BRAF*^ cell lines, respectively (Fig. 1d). Induced expression of BRAF(V600E) mutant protein in SK-MEL-119^NRAS*^, WM1361^NRAS*^, SK-MEL-63^NRAS*^ cells and of NRAS(Q61R) mutant protein in MGH-CH-1, GMEL and A375 cells were all confirmed by western blotting at sixth day of cell viability (Fig. 1b−d). Figure S3 shows the reduction in cellular density and morphologic changes associated with rival oncogene overexpression in antagonistic lines but not in neutral cell lines at day 5. We next examined the cellular response to oncogene antagonism. Forced expression of the rival oncogene in SK-MEL-119^NRAS*^, WM1361^NRAS*^ and GMEL cells led to steep increases in the percentage of SA-ß-gal-positive cells (Fig. 2a, upper panel). This senescence response was notably absent in the neutral SK-MEL-63 and A375^BRAF*^ lines (Fig. 2a, lower panel). In cell cycle analyses, secondary oncogene induction led to cell cycle arrest at different phases such as G2/M arrest in both SK- MEL-119^NRAS*^ + iBRAF* (1.5-fold, *p* < 0.01) and GMEL^BRAF*^ + iNRAS* (1.63-fold, p < 0.01) lines and G2/M arrest in the WM1361^NRAS*^ (1.44-fold, *p* < 0.01) line; there was no significant evidence of arrest in the SK-MEL-63^NRAS*^ + iBRAF* or A375^BRAF*^ + iNRAS* cells (Fig. 2b). Though there were no significant changes in subG1 populations, FACS analysis with Annexin-V staining revealed significant increases in the percentage of apoptotic cells in two NRAS(Q61R) lines (SK-MEL-119^NRAS*^ + iBRAF* and WM1361^NRAS*^ + iBRAF*) but not in the vulnerable GMEL^BRAF*^ + iNRAS* line or the neutral SK-MEL-63^NRAS*^ + iBRAF* and A375^BRAF*^ + iNRAS* lines (Fig. 2c). Dual oncogene expression also reduced long-term self-renewal capacity (2 weeks) as introduction of the rival oncogene suppressed colony formation in the sensitive SK-MEL-119^NRAS*^ + iBRAF*, WM1361^NRAS*^ + iBRAF* and GMEL^BRAF*^ + iNRAS* lines but not the neutral SK-MEL-63^NRAS*^ + iBRAF* and A375^BRAF^* + iNRAS* lines (Fig. 2d). These data suggest that mixed inhibitory inputs including cell cycle arrest, apoptosis and eventual senescence (“secondary OIS”) may all contribute in part to the observed oncogene exclusion. It also suggests a diverse but possibly coordinated effort to halt cellular expansion. Thus, we next sought to elucidate potential mediators of this dual oncogene antagonism.

### Molecular response to dual oncogenesis

Examination of signaling cascades which might be activated in response to dual oncogene expression was not initially revealing. There were some modest and inconsistent increases in both pMEK and pERK, which did not appear to correlate with the observed response (Fig. S4). Even when broader signal profiles were obtained with phosphokinase arrays, no recurrent phosphorylation events were noted (data not shown). These findings indicate that signaling differences may not underpin the antagonistic phenotype, but rather, the molecular circuitry may itself be reprogrammed. Thus, we set out to map the molecular events which are downstream of dual oncogenesis.

**Fig. 3:**
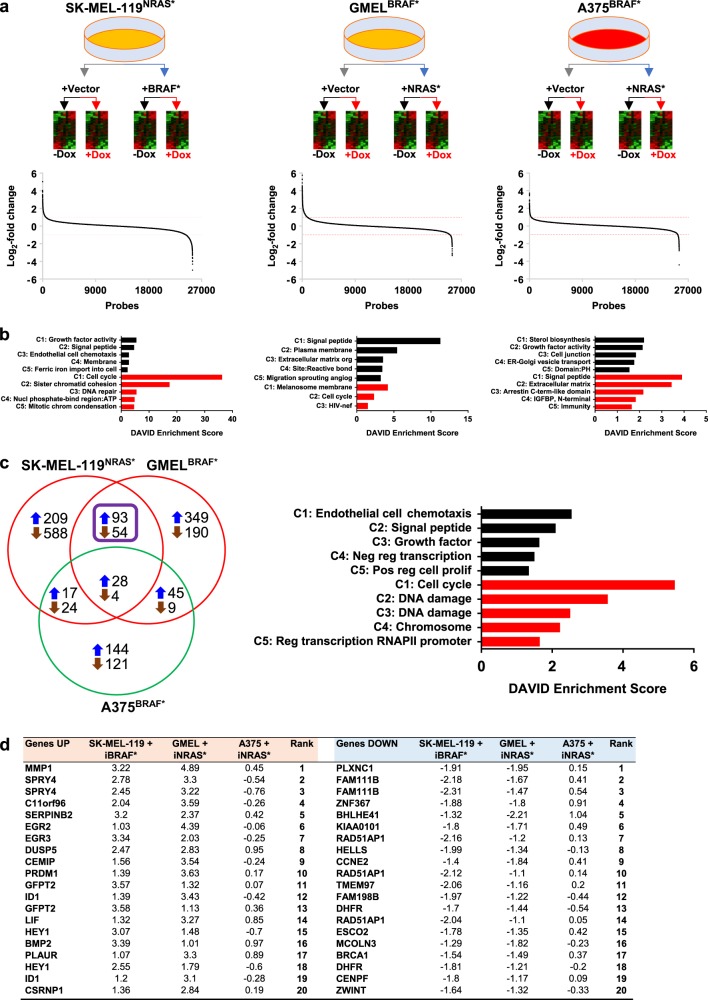
Rival oncogene upregulates SPRY4 expression in growth suppressive melanoma cells. **a** The overall workflow and heat map results of microarray analysis of mRNA isolated from three melanoma cell lines as indicated. log2-fold differences as obtained by [log2(+Dox/oncogene) − log2(no Dox/oncogene)] − [log2(+Dox/vector) − log2(no Dox/vector)]. **b** DAVID enrichment scores (ES) for set of all genes that were significantly increased or decreased by at least twofold among antagonistic; SK-MEL-119^NRAS*^ + iBRAF*, GMEL^BRAF*^ + iNRAS* and neutral; A375^BRAF*^ + iNRAS* cell lines. **c** DAVID enrichment of growth suppressive overlapping significantly up- and downregulated (=2 folds) genes in SK-MEL-119^NRAS*^ + iBRAF*, GMEL^BRAF*^ + iNRAS* cell lines but not A375. **d** *SPRY4* transcripts were among the most upregulated ones in both SK-MEL-119^NRAS*^ and Gmel^BRAF*^, but not A375^BRAF*^ cell lines

To identify genes involved in mediating oncogene antagonism, we performed a comparative genome-wide expression (GEX) analysis using a design outlined in Fig. 3a. Since the dual oncogenesis occurs in isogenically matched lines, we first examined expression changes in paired analyses (Table S1). In the SK-MEL-119^NRAS*^ + iBRAF* line, 2.20 and 4.43% of the gene probes were upregulated (i.e. increased 2-fold; >1.0 log2-fold) and downregulated (i.e. decreased 2-fold; <−1.0 log2-fold), respectively, upon induction of exogenous *BRAF*(V600E). These were 3.44 and 1.39% up- and downregulated, respectively, for GMEL^BRAF*^ + iNRAS* and 1.24 and 0.89% up- and downregulated, respectively, for A375^BRAF*^ + iNRAS*. With BRAF* overexpression in SK- MEL-119^NRAS*^ + iBRAF*, the most induced genes were *IL1B* (5.04 log2-fold; Table S1), *MMP1* (4.04 log2-fold), *IL1A* (3.93 log2-fold), *IL24* (3.84 log2-fold) and *GFPT2* (3.58 and 3.57 log2-fold) while the most suppressed genes were in *CXL12* (−4.99, −3.66 and −3.62 log2-fold), *MGP* (−4.31 log2-fold), *CHRNA1* (−3.95 and −3.58 log2-fold), *PLPPR4* (−3.7 log2-fold) and *TRIM22* (−3.55 log2-fold). With the introduction of NRAS* in GMEL^BRAF*^ + iNRAS*, the most upregulated genes were *STC1* (5.75 log2-fold), *CXCL8* (5.3 log2-fold), *MMP1* (4.89 log2-fold), *IGFBP3* (4.55 log2-fold) and *MB* (4.43 log2-fold) and the most downregulated genes were *TRIM63* (−3.34 log2-fold), *PHACTRI* (−3.18 log2-fold), *MLANA* (−3.07 log2-fold), *TYRP1* (−2.82 log2-fold) and *GAGE* genes (−2.61 log2-fold). In contrast, for the neutral A375^BRAF*^ + iNRAS* line, *GDF15* (3.75, 3.58, 3.45 log2-fold), *PTPRR* (2.95 log2-fold), *UCA1* (2.86 log2-fold), *STC1* (2.83 log2-fold) and *SLC14A1* (2.61 log2-fold) exhibited the greatest increase while *MGP* (−4.4 log2-fold), *ITGA9* (−2.81 log2-fold), *SERPINF1* (−2.66 log2-fold), *A2M* (−2.58 log2-fold) and *ENPP2* (−2.54, −2.48 log2-fold) exhibited the most profound decrease in expression levels. We next subjected the set of all genes that were increased or decreased by at least twofold to functional clustering using DAVID (Fig. 3b and Tables S2−S4). Among upregulated genes (i.e. >2-fold), the “SIGNAL PEPTIDE” functional cluster was the highest and second highest annotated cluster in GMEL^BRAF*^ + iNRAS* (Enrichment score, ES:11.26) and SK-MEL-119^NRAS*^ + iBRAF* (ES:4.62), respectively. Interestingly, the “SIGNAL PEPTIDE” cluster was the leading annotated set among the most suppressed genes for A375^BRAF*^ (ES: 3.91). The “CELL CYCLE” functional cluster ranked first and second in enrichment, among the set of most suppressed genes (i.e. >2-fold), in the SK-MEL-119^NRAS*^ + iBRAF* (ES: 36.26) and GMEL^BRAF*^ + iNRAS* lines (ES: 2.32), respectively; the “CELL CYCLE” cluster were not significantly enriched in the A375^BRAF*^ + iNRAS* line. One notable cluster is “MELANOSOME MEMBRANE”, which was derived from the set of most downregulated genes (i.e. >2-fold) in 18 GMEL^BRAF*^ + iNRAS* (ES:4.18). To replicate this finding, we used a published list of high impact MITF target genes [9], MITF targets, as a group, were significantly more suppressed than non-MITF targets (Fig. S6b); log2-fold −0.56 ± 0.03 vs. −0.0034 ± 0.0032, *p* < 0.0001, Student’s *t* test) in GMEL^BRAF*^ cell lines. In addition, *MITF* suppression was verified by qPCR in the GMEL^BRAF*^ + iNRAS* cell lines (Fig. S6a). Thus, lineage programming appears to be attenuated in the GMEL^BRAF*^ + iNRAS* line with ectopic NRAS* expression. Lastly, in only the A375^BRAF*^ + iNRAS* cells, overexpression of NRAS* appears to correlate with a functional cluster related to reprogramming of lipid metabolism (“STEROL BIOSYNTHESIS”, ES:2.2).

We next sought to identify a shared suppressive physiology by focusing on statistically significant regulated genes in both SK-MEL-119^NRAS*^ + iBRAF* and GMEL^BRAF*^ + iNRAS* lines but not the A375^BRAF*^ + iNRAS* line. As shown in Fig. 3c, there were 93 upregulated transcripts (i.e. >2-fold increase) shared between SK-MEL-119^NRAS*^ + iBRAF* and GMEL^BRAF*^ + iNRAS* upon induction of the rival oncogene while there were 54 downregulated (i.e. >2-fold decrease) transcripts shared by these two antagonized lines. For the set of altered genes shared by the two lines, the “CELL CYCLE” functional cluster (Table S5) showed the greatest enrichment among the most downregulated genes (i.e. >2-fold decrease; ES: 5.46) followed by two DNA damage clusters (ES:3.57 and ES: 2.51). Among the most upregulated genes (i.e. >2-fold increase) shared by SK-MEL-119^NRAS*^ + iBRAF* and GMEL^BRAF*^ + iNRAS* but not A375^BRAF*^ + iNRAS*, the “ENDOTHELIAL CELL CHEMOTAXIS” (ES: 2.55) cluster, a ‘SIGNAL PEPTIDE” (ES: 2.1) cluster and the “GROWTH FACTOR” (ES: 1.64) cluster exhibited the strongest enrichments.

While it is likely that many concurrent pathways have been activated to bring about growth arrest in the SK-MEL-119^NRAS*^ + iBRAF* and GMEL^BRAF*^ + iNRAS*, *SPRY4* transcripts were among the most upregulated ones in both antagonized SK-MEL-119^NRAS*^ + iBRAF* and GMEL^BRAF*^ + iNRAS* lines, but not in the neutral A375^BRAF*^ + iNRAS* cell line (Fig. 3d Table). As *SPRY4* has been implicated as a tumor suppressor, we set out to explore the possibility that SPRY4 is mediating growth suppression selectively in the antagonistic lines.

### SPRY4 as a negative regulator of melanoma growth and mediator of oncogene antagonism

**Fig. 4:**
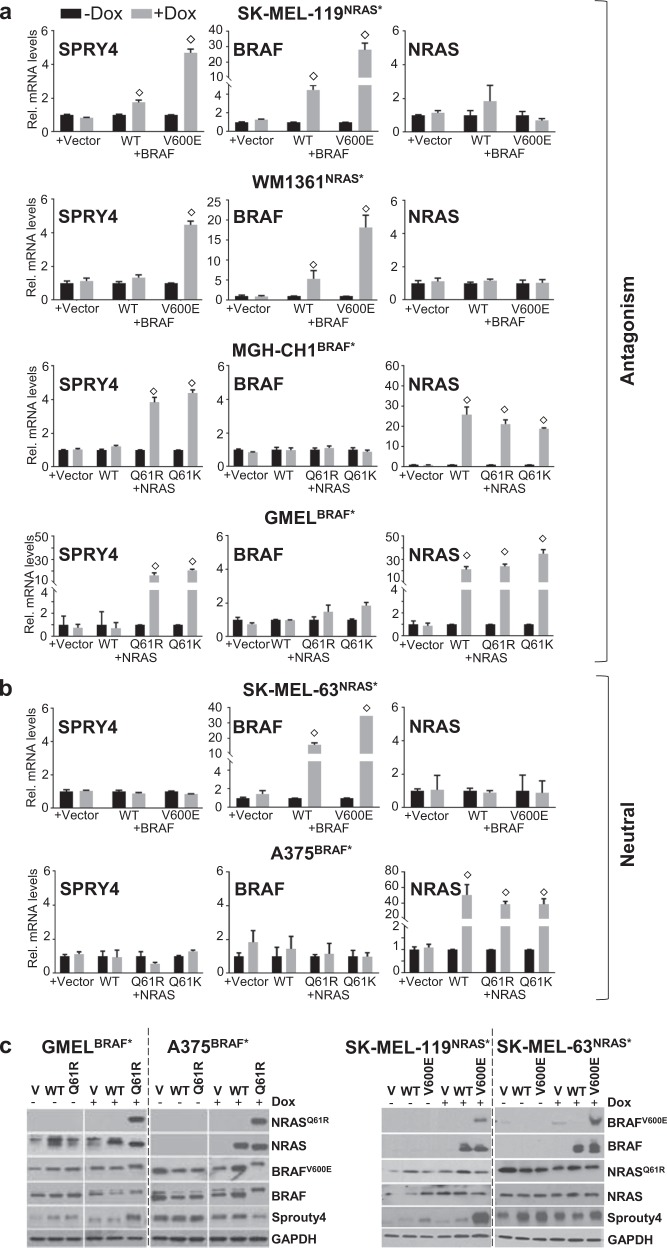
SPRY4 as a mediator of oncogene antagonism. **a** qPCR validating *SPRY4* upregulation upon rival oncogene expression in all the four cell lines showing antagonism (SK- MEL-119^NRAS*^, WM1361^NRAS*^, MGH-CH-1^BRAF*^ and GMEL^BRAF*^ but **b** not the neutral SK-MEL-63^NRAS*^ and A375^BRAF*^ cell lines. After normalization to *GUSB1* RNA, data were presented as mean ± SD of triplicates experiment. **c** Parallel increases in the protein level was also confirmed in two suppressive cell lines but not in two neutral cell lines after induction with/without 50–100 ng/ml of doxycycline for 2–3 days by western blotting. Student’s *t* test, doxycycline vs. no-doxycycline, ^◇^*p* *≤* 0.05. The data presented are representative of three independent experiments

*SPRY4* induction was first corroborated by qPCR in all four lines exhibiting antagonism (i.e. SK-MEL-119^NRAS*^ + iBRAF*, WM1361^NRAS*^ + iBRAF*, MGH-CH-1^BRAF*^ + iNRAS* and GMEL^BRAF*^ + iNRAS* lines; Fig. 4a) but in none of the neutral lines (i.e. SK-MEL-63^NRAS*^ + iBRAF* and A375^BRAF*^ + iNRAS*; Fig. 4b). At the protein level, there were similar increases in SPRY4 in the two suppressible lines (SK-MEL-119^NRAS*^ + iBRAF* and GMEL^BRAF*^ + iNRAS*) but not in two nonsuppressed lines (SK-MEL-63-NRAS^Q61K^ + iBRAF* and A375^BRAF*^ + iNRAS*) (Fig. 4c); interestingly, there was a notable decrease of SPRY4 in the A375^BRAF*^ + iNRAS* line. These results verify the microarray findings and corroborate the phenotypic correlation between SPRY4 and oncogene antagonism.

**Fig. 5:**
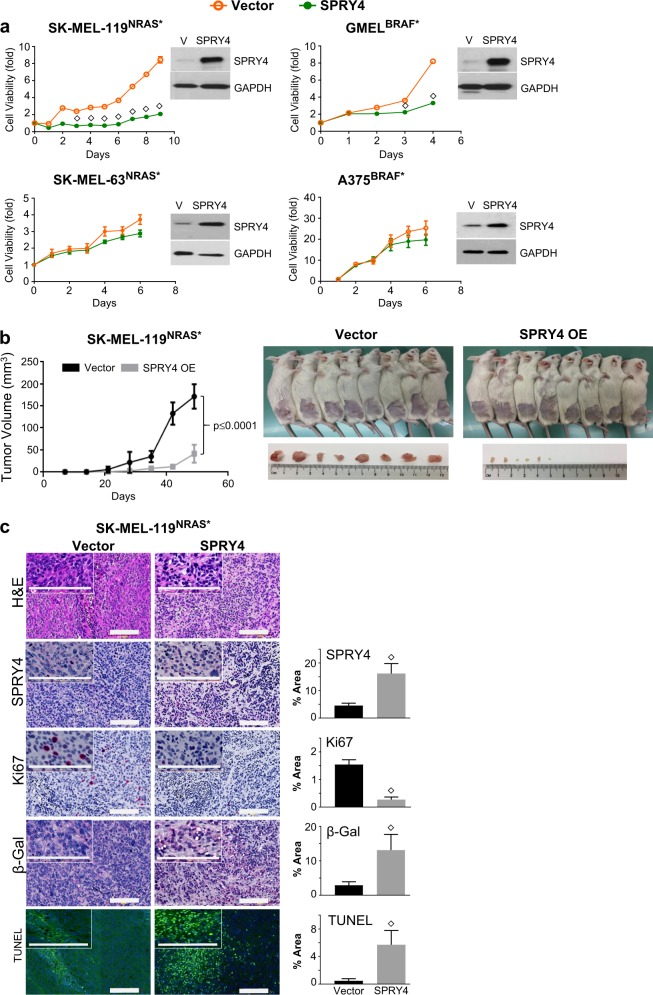
*SPRY4* overexpression inhibits in vitro and in vivo cell growth. **a** Following third day of *SPRY4* overexpression, two antagonistic lines, SK-MEL-119^NRAS*^, GMEL^BRAF*^ and two neutral lines, SK-MEL-63^NRAS^* and A375^BRAF*^, were subjected for 6 days cell viability assays. Also, SK-MEL-119^NRAS*^ cells were subcutaneously injected in NSG mice at 0.2 million per flank per animal into 8 mice per group. **b** Tumor growth curve and tumor burden tolerated by mice was measured every seventh day for 7 weeks and plotted (mean ± SD, *N* = 8). **c** A representative image of H&E and IHC in 5 µm sections of xenograft tumors. Protein levels of SPRY4, positive staining of SA-ß-galactosidase in cytoplasm, and Ki-67 mainly detected in the nuclei. TUNEL-positive cells significantly increased in SPRY4 overexpressed tumors. Student’s *t* test, vector vs. SPRY4, ◇*p* ≤ 0.05. Bar, 150 µm. Measurement of percentage area and pixel value statistics of three defined selections covered by the stained cells using analyze tool in ImageJ software

To more directly prove that SPRY4 is mediating the synthetic suppression, we set out to determine if SPRY4 itself is growth suppressive, both in vitro and in vivo, and if the depletion of *SPRY4* can rescue cells from the observed antagonism. As shown in Fig. 5a, constitutive expression of *SPRY4* dramatically inhibited proliferation of SK- MEL-119^NRAS*^ and GMEL^BRAF*^ cells (upper panel) but not in neutral A375^BRAF*^ and SK-MEL-63^NRAS*^ cells (lower panel). In vitro analysis of SK-MEL-119^NRAS*^ confirmed the increase in apoptosis and SA-ß-galactosidase enzyme activity with SPRY4 induction (Fig. S5a). Figure S5b shows the reduction in density along with morphologic changes associated with SPRY4 overexpression (day 5).

**Fig. 6:**
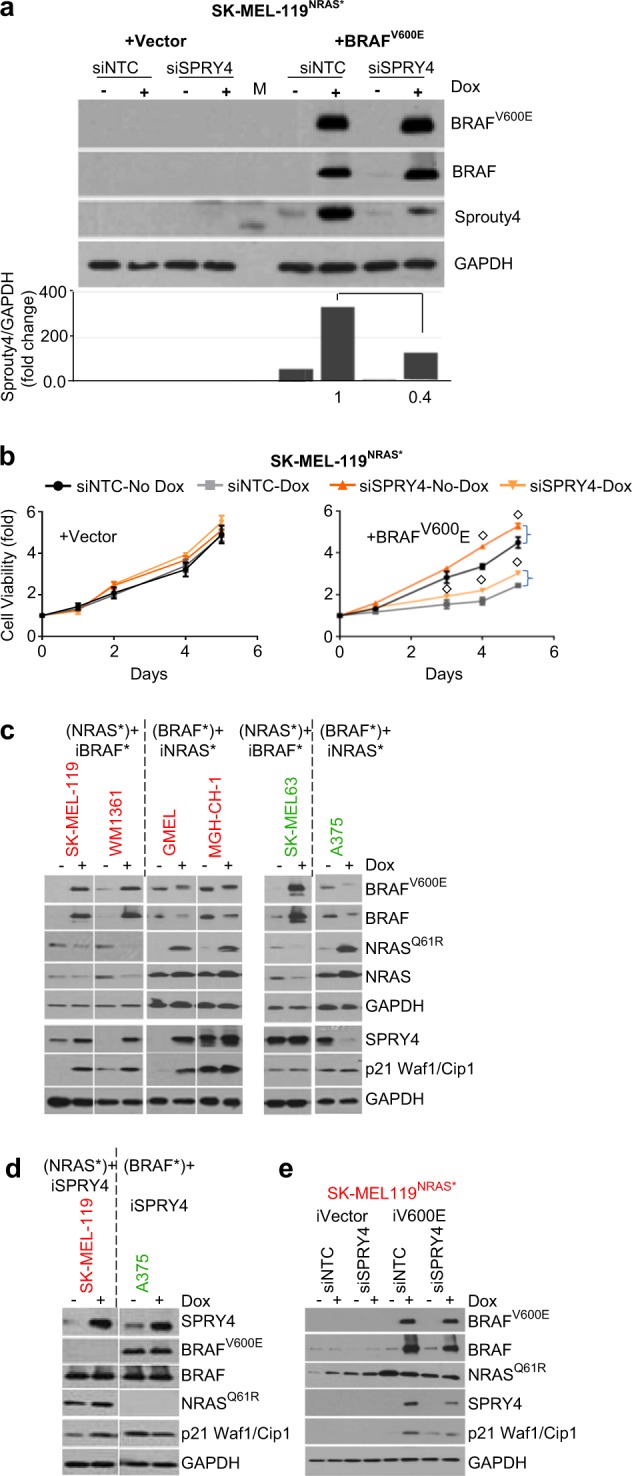
Rival oncogene-induced growth suppression is rescued by *SPRY4* silencing in vitro. **a** Western blot analysis and quantitative densitometry of the protein expression in SK- MEL-119^NRAS*^ + iBRAF*cells that ectopically express rival oncogenes and siRNA SPRY4 constructs. Total cell lysate extracts at 48 h were probed with antibodies for BRAF(V600E), BRAF(WT), NRAS(Q61R), NRAS(WT), SPRY4 and internal loading control GAPDH. **b** Silencing of *SPRY4* abrogates the growth inhibition effect of rival oncogene expression in SK-MEL-119^NRAS*^ cells as compared to siRNA nontarget control (siNTC) vector. **c** Differential regulation of SPRY4 and p21 proteins upon second oncogene induction. **d** *SPRY4* overexpression and (**e**) SPRY4 silencing effect on p21 protein expression in the presence of rival oncogene, among suppressive (Red) and nonsuppressive (Green) cell lines was confirmed by western blotting. Student’s *t* test, doxycycline vs. no-doxycycline, ^◇^*p* *≤* 0.05. The data presented are representative of three independent experiments

To further investigate whether SPRY4 overexpression can reduce tumor growth in vivo, SK-MEL-119^NRAS*^ cells were transfected either with empty CD516B-2-Vector or CD516B-2-SPRY4 and subjected to xenograft experiments. As shown in Fig. 5b, SPRY4 profoundly inhibited tumorigenesis in NSG mice (*p* < 0.0001) with no change in body weight (Fig. S5c). Histologically, tumors that overexpressed SPRY4 had reduced overall cellularity and increased fibrous stroma (Fig. 5c). Compared to control tumors, SPRY4 tumors also harbored reduced Ki67, enhanced tumor cell apoptosis as shown by TUNEL staining and increased SA-ß-gal expression (Fig. 5c). Taken together, SPRY4 appears to suppress in vivo tumor xenograft growth, which can be partially explained by an increase in senescence similar to the observations in vitro. We next sought evidence of rescue from oncogene antagonism by depleting SPRY4 in SK-MEL-119^NRAS*^ + iBRAF* cells using siRNA’s against *SPRY4*. Effective SPRY4 suppression by siRNA in SK-MEL-119^NRAS*^ + iBRAF* (2.47-fold decrease) and rival oncogene induction by doxcycyline lines were confirmed by western blotting (Fig. 6a). Phenotypically, in the SK-MEL-119^NRAS*^ + iVector i.e. Tet-On-vector control (Fig. 6b; left panel) cells, SPRY4 depletion alone had minimal effects on proliferation as growth in the siNTC and siSPRY4 lines were similar in the SK-MEL-119^NRAS*^ + iVector control. However, upon induction of iBRAF* in SK-MEL-119^NRAS*^, there was a significant arrest, as expected, with the control siNTC (Fig. 6b, black circle line to gray box line). When *SPRY4* was additionally depleted with siSPRY4, there was a significant rescue (Fig. 6b, gray box line to yellow inverted triangle line) from iBRAF*-mediated suppression. These results support the idea that SPRY4 can, at least in part, mediate oncogene antagonism.

Lastly, we set out to determine if SPRY4 could be linked to a marker of senescence, p21 Waf1/Cip1. Dual BRAF* + NRAS* expression upregulated p21 in three out of the four suppressed lines but not in either of the neutral lines (Fig. 6c). We next generated a tetracycline-inducible SPRY4 cassette in SK-MEL-119^NRAS*^ and A375^BRAF*^ lines (Fig. 6d) and showed that dox-mediated expression of SPRY4 led to increased p21 in the susceptible SK-MEL-119^NRAS*^ line but not the neutral A375^BRAF*^ line. Moreover, suppression of SPRY4 with siSPRY4 in the SK-MEL-119^NRAS*^ line partially abrogated the p21 increase which accompanied iBRAF* (Fig. 6e). These findings are consistent with p21 as one of the factors which is downstream of SPRY4 and which may contribute to secondary OIS.

## Discussion

Deep tumor sequencing has uncovered a myriad of mutational signatures and patterns [10, 11]. Although *BRAF* and *NRAS* are frequently mutated in human melanoma, coexistence of *BRAF* c.1799T>A(V600E) and *NRAS* c.181C>A (Q61K)/182A>G (Q61R) changes within the same melanoma tumor is essentially nonexistent [2, 12] except in situations of acquired resistance to BRAF inhibitors [3]. Despite the widespread recognition of this exclusive relationship, the evolution of these innate patterns as dictated by tumor, host and environmental forces remains largely unknown. The overall objective of our studies is to better understand the underlying pathophysiology which could explain the well-established exclusivity between BRAF(V600E) and NRAS(Q61) mutations in melanoma. We primarily focused on “oncogene antagonism” since mechanistically, a trimming of double mutants would be most compatible with observed mutational pattern in vivo.

Through our analysis, we identified *SPRY4* as one possible mediator of oncogene antagonism. This is supported by several lines of evidence including the (i) upregulation of SPRY4 by the rival oncogene only in cell lines exhibiting suppression and in none of the nonsuppressed lines, (ii) stronger growth inhibitory effects of SPRY4 in the antagonistic compared to neutral lines, (iii) direct tumor suppression in vivo by SPRY4 and (iv) partial rescue from oncogene antagonism with depletion of *SPRY4*. SPRYs and SPREDs comprise a family of proteins which are engaged in a negative regulatory loop in that both are activated by, and serve to repress, MAPK signaling [13]. SPRY4 has been shown to directly bind to and inhibit RAF1 and BRAF(WT), but not BRAF*, through the carboxy-terminal cysteine-rich domain [14, 15]. Since our cell lines all harbor BRAF*, either as an innate mutation or as the rival oncogene, MAPK signaling in our experimental conditions may be functionally resistant to SPRY4-mediated suppression. Therefore, the precise molecular mechanism by which SPRY4 mediates synthetic suppression remains to be clarified but may involve upregulation of p21.

The contrasting effects of NRAS* on two distinct BRAF* lines are also worth noting. In the synthetically suppressed GMEL^BRAF*^ + iNRAS* cells, there is a dramatic suppression of *MITF* and its targets (Fig. S6). Melanoma cells have been shown to be “addicted” to MITF [16] and thus the NRAS*-mediated downregulation of MITF could cooperate with the upregulation of SPRY4 to suppress growth. In oncogene-resistant A375^BRAF*^ cells, sterol biosynthesis appears to be activated in the context of dual oncogenesis. Both glycolytic and lipid metabolic reprogramming is now well established in cancer and is thought to allow malignant cells to adapt to a hostile microenvironment [17]. The secondary acquisition of an NRAS* mutation in a BRAF* melanoma cell has also been described in the context of therapeutic resistance [18]. In both the A375^BRAF*^ + iNRAS* and MGH-CH-1^BRAF*^ + iNRAS* lines, we did observe resistance to BRAF inhibition (Fig. S7), suggesting that resistance can be engendered de novo even in the absence of drug.

While there were detectable effects on the cell cycle and on the apoptotic response, the dual NRAS*/BRAF* mutant state is associated with an increase in cells with SA-ß-gal and a decrease in colony formation, both of which correlate with heightened cellular senescence. We interrogated our microarray data using the GSEA Fridman senescence gene set (Fig. S8) and found that only GMEL^BRAF*^ + iNRAS* exhibited a significantly higher level of induction of the senescence gene set relative to all other transcripts (mean log2-fold +0.068 ± 0.025 vs. −0.012 ± 0.003, *p* < 0.0001). (http://software.broadinstitute.org/gsea/msigdb/cards/FRIDMAN_SENESCENCE_UP.html) These results echo a prior study in which Petti and colleagues overexpressed NRAS(Q61R) into a single BRAF(V600E) melanoma line and similarly observed the eventual onset of a senescence phenotype [6]. While oncogenes have been shown to induce senescence in primary cells [19, 20], NRAS* and BRAF*-mutated melanoma cells have theoretically negotiated this checkpoint to attain a malignant and immortal state. Thus, oncogene antagonism appears distinct from primary OIS and may be more appropriately termed “secondary” OIS. One senescence-associated gene worth mentioning is *CDKN1A* (p21). This cell cycle inhibitor is upregulated in senescent cells [21] and we were able to show that the protein is increased in three of the four suppressed lines with the introduction of the rival oncogene. Moreover, induction and suppression of *SPRY4* by genetic means selectively upregulated and downregulated p21, respectively. Sprouty4 has been reported to reduce cell growth in NSCLC cells by upregulating the expression of tumor suppressor p21 [22]. These data suggest a possible mechanism whereby NRAS* + BRAF* increases SPRY4, which in turn stimulates p21 and secondary OIS (Fig. S10).

Petti et al. [6] and Tuyn et al. [23] also noted an increase in immune recognition and antigen processing machinery with OIS though in the setting of primary melanocytes. At the RNA level for the MHC molecules, both SK-MEL-119^NRAS*^ + iBRAF* and A375^BRAF*^ + iNRAS* demonstrated significant decreases (*p* < 0.0001 for both) in HLA-D with variable smaller level changes in HLA-A/B/C/E/F (Fig. S9). In contrast, GMEL^BRAF*^ + iNRAS* cells had a significant upregulation of the nonclassical MHC-I, HLA-E (*p* = 0.012), which has been shown to be increased during replicative senescence [24]. With GO functional analysis, the “ANTIGEN PROCESSING AND PRESENTATION OF EXOGENOUS PEPTIDE ANTIGEN” cluster (ES: 4.63) was enriched among the most downregulated genes (i.e. >2-fold decreased) in SK-MEL-119^NRAS*^ + iBRAF* (Table S2) while the “IMMUNITY” (ES: 1.65) and “TYPE-1-IFN SIGNALING PATHWAY” (ES: 1.52) clusters exhibited weaker enrichment among the most downregulated genes (i.e. >2-fold decreased) in the A375^BRAF*^ + iNRAS* cells (Table S4). In GMEL^BRAF*^ + iNRAS*, the “CYTOKINE-CYTOKINE RECEPTOR” cluster (ES: 2.49) was significantly enriched among the most upregulated genes (i.e. >2-fold increased, Table S3). These disparate lines of evidence suggest a complex interaction between oncogene, senescence and immune surveillance.

There are inherent limitations to our analyses. First, the relationship between our in vitro results and in vivo human findings remain mostly inferential since there is a lack of sufficient cohorts of dual mutant tumors to verify our results. Second, our experiments span from days to weeks but are not considered long term. Thus, slow but persistent selection against double mutants may not be detected in some of the synthetic neutral lines. Third, while we provide evidence that SPRY4 may mediate some of the synthetic suppression, it is unlikely to be the only inhibitory mediator. Other upregulated genes (e.g. ID1, DUSP5) have also been implicated in growth arresting physiologies such as senescence [25, 26]. Lastly, given our biologic interest in oncogene exclusion, we have not fully examined the genetic and/or molecular differences along with immune surveillance which dictate sensitivity or resistance to the acquisition of a rival oncogene.

In conclusion, we have uncovered a range of proliferative responses to dual BRAF* and NRAS* expression in melanoma lines. Synthetic suppression could be observed in a subset of lines and could explain the clinical phenomenon of mutual exclusivity. Through comparative molecular profiling, we identified SPRY4 as a potential mediator of the arrested physiology, though diverse biologic pathways appear to be engaged in the process. As many cells exhibit markers of senescence with oncogene antagonism despite the absence of traditional gatekeepers of OIS, “secondary” OIS may in fact play a role in the evolution of mutation patterns.

## Materials and methods

### Molecular cloning

Following assays were conducted as previously described by our laboratory [27, 28]. Lentiviral particles were used to deliver tetracycline-inducible recombinant DNA constructs. Full-length BRAF(WT) and BRAF(V600E) were PCR amplified from pBABE-B-Raf, pBABE-B-Raf-V600E vector (Addgene) using primers (Forward: 5′- ATATGGCCCCCGGGGACGCGTGCCATGGCGGCGCTGAGC-3′ and Reverse: 5′- TCCCCTACCCGGTAGAATTCTCAGTGGACAGGAAACGCAC-3′) with amplicon possessing the t1799a/v600e mutation. Similarly, pBABE-NRAS(WT), pBABE-NRAS(Q61R), and pBABE-NRAS(Q61K) (Addgene) (Forward: 5′- ATATGGCCCCCGGGGACGCGTGCCATGACTGAGTACAAACTGGTG-3′, Reverse: 5′-TCCCCTACCCGGTAGAATTCTTACATCACCACACATGGC-3′) and human SPRY4 (Origene) (Forward: 5′-CCCGGACGCGTGCCATGCTCAGCCCCCTC-3′ and Reverse: 5′-TACCCGGTAGAATTCTCAGAAAGGCTTGTCGG-3′) vectors were used to PCR amplify respective genes and cloned in lentiviral Tet-On pLVX-TRE3G-ZsGreen1 (Clontech) and constitutive CD516B-2 (System Biosciences, Palo Alto, CA) vectors. The ligation product was transformed into competent *Escherichia coli* bacterial strain DH5a (NEB). The empty vector (pLVX-TRE3G-ZsGreen1) designated as “Tet-On vector” and overexpressing recombinant constructs (pLVX-TRE3G-ZsGreen1-GENE), designated as “Tet-On GENE”, were used to generate stable cell lines.

### Lentiviral production and generation of Tet-inducible table melanoma cell lines

293FT (ATCC, Manassas, VA) cells were plated in 60 mm dish at 3 × 10^6^ cells/plate. After 24 h, cells were transfected with 3.5 µg of target lentiviral construct, along with 17 µl Lenti-X HTX packaging Mix2 using 3.75 µl Xfect polymer and incubated overnight. The next day, fresh media was added, at 48 h of post-transfection, supernatant was harvested and centrifuged briefly at 500 × *g* for 10 min to remove cellular debris as per the manufacturer’s instructions (Clontech). Virus-containing media was stored at −80 °C until infection. A day prior of transduction, stable regulator (pLVX- EF1a-TET3G vector, G418 (0.5–2 mg/ml)) melanoma cells were plated at density 3 × 10^5^ cells per well with 2 ml Dulbecco's Modified Eagle Medium (DMEM) (containing 10% tetracycline-free fetal bovine serum (FBS), designated as Tet-free medium) in six-well plate. Next day, lentivirus supernatant of 0.1−0.2 ml of 5 ml (MOI:1.67) and 8 µg/ml of polybrene were added over cells containing fresh Tet-free medium in a total volume of 1 ml. Cells were incubated overnight at 37 °C in humidified 5% CO_2_ incubator before replacing with 2 ml fresh Tet-free medium without antibiotics for expansion of the transductants. Cells were selected with puromycin at 2–6 µM for another 15 days before further experiments. The stable clones were propagated, and protein expression levels were confirmed with western blotting.

### Determination of the transcript copy number of BRAF and NRAS and its comparison to endogenous levels

Total mRNA was collected from doxycycline-induced and noninduced melanoma cell lines and 2 µg RNA was converted to cDNA (High Capacity RNA-to-cDNA Kit). The Wagatsuma method [29] was used to determine the exact transcript copy numbers of *BRAF* and *NRAS*, both of which was compared with two standard curves plotted with the threshold cycles (Cq values) from recombinant plasmid (rDNA) and sample cDNA diluted 1/10 five serial dilutions. The standards of sample cDNA are from Tet-On BRAF(V600E) SK-MEL-119^NRAS*^ designated as “SK-MEL-119^NRAS*^ + iBRAF*”, or Tet-On NRAS(Q61R) GMEL^BRAF*^ designated as “GMEL^BRAF*^ + iNRAS*” cell lines induced with doxycycline 100 ng/ml for 3 days. We first obtained the standard curves of *BRAF* and *NRAS* using recombinant plasmids (pcDNA-NRASQ61R or pcDNA-BRAFV600E) solutions. The copy numbers of recombinant plasmid (rDNA) was within the range of 101−105 copies per reaction, and the standard curve from SK-MEL-119^NRAS*^ + iBRAF* or from GMEL^BRAF*^ + iNRAS* cDNA was parallel (arbitrary units) to their corresponding recombinant plasmid standard curve (Fig. S1a, b). The standard curve slopes/E-amp (amplification efficiency) for the two target genes were (BRAF: −3.2377/2.036 for the recombinant plasmid (rDNA) standard and −3.3810/1.976 for the sample cDNA solutions and NRAS: −2.9053/2.209 for the recombinant plasmid (rDNA) standard and −3.4705/1.941 for the sample cDNA solutions). These results confirm that samples amplify target BRAF and NRAS nearly at same efficiency as rDNA plasmid solutions, respectively.

To get transcript copy numbers, we took the efficiencies of both the standards into an account and calculated the copy number of mRNA [29, 30]. Next, we determined the transcript copy number of *BRAF* and *NRAS* mRNA in a broader panel of cell lines and doxycycline induced or noninduced SK-MEL-119^NRAS*^ + iBRAF* and GMEL^BRAF*^ + iNRAS* melanoma cells (Fig. S1c, d). The mRNA copies per micro liter of the sample solution at 0−1000 ng/ml doxycycline at third day was determined to range from 1.54 × 10^6^ to 99.97 × 10^6^ for BRAF mRNA in SK-MEL-119^NRAS*^ + iBRAF* and  81.047× 10^6^ to 4847.11 × 10^6^ for NRAS mRNA in GMEL^BRAF*^ + iNRAS* and RNA expression found to be well sustained in all the melanoma lines. Hence, by calculating the average value of triplicate samples at 50−100 ng/ml doxycycline in respective cell lines, the transcript copy number after induction for BRAF was from 27.66 × 10^6^ to 42.48 × 10^6^ in SK-MEL-119^NRAS*^ + iBRAF* which is nearly parallel to A373-C6 and K2 cells and for NRAS was from 1373.25 × 10^6^ to 2092.42 × 10^6^ which is almost parallel to A373-C6 and SK-MEL-28 cell lines. Thus, the mRNA copy number obtained with 50−100 ng/ml doxycycline were close to naïve cell lines. Therefore, the induction was in physiological range and comparable to endogenous level of many melanoma cell line (Fig. S1c, d).

### Establishing stable melanoma cell lines

Construction of stably expressing genetically homogenous stable cell lines such as SK-MEL-119^NRASQ61R^ + iBRAF(V600E), WM-1361^NRASQ61R^ + iBRAF(V600E), GMEL^BRAFV600E^ + iNRAS(Q61R), MGH-CH-1^BRAFV600E^ + iNRAS(Q61R), SK-MEL-63^NRASQ61K^ + iBRAF(V600E) and A375^BRAFV600E^ + iNRAS(Q61R), MGH-SW-1^NRASQ61K^ + iBRAF(V600E), SK- MEL-5^BRAFV600E^ + iNRAS(Q61R), WM164^BRAF^ + iNRAS(Q61R), Pmel + iNRAS*/iBRAF* infected with lentivirus were either selected with antibiotic puromycin (2–6 µg/ml) for 2 weeks or sorted by FACS (FACS Aria (BD Biosciences-US) with 0.05% trypsin-Ethylenediaminetetraacetic acid (EDTA) solution and resuspended at 1 × 10^6^ cells/ml Tet-free growth medium (Clontech). A total of vital ~1 × 10^5^ green fluorescent protein cells were collected, grown and protein expression analyzed by western blotting before performing assays.

### Western blot analysis and phospho-kinase profile

Cells with indicated conditions (Doxycycline: +Dox, 50–100 ng/ml or No-Doxycycline: -Dox) were screen harvested, washed with 1× phosphate-buffered saline and lysed in a RIPA buffer (Boston Bioproducts, Ashland, MA) supplemented with halt protease inhibitor cocktail (Thermo Scientific, Rockford, IL). The protein concentrations of the lysates were measured by BCA protein assay kit (Bio-Rad, Hercules, CA). The lysates (10–20 µg protein) were separated on 4–20% SDS polyacrylamide mini-gels (Bio-Rad, Hercules, CA) and transferred to PVDF or nitrocellulose membrane followed by western blot analysis as described previously [27]. The antibodies used were as follows: mouse monoclonal anti-BRAF(V600E) (1:4000, Spring Bioscience, Pleasanton, CA), NRAS(Q61R) (1:2000, Abnova, Atlanta, GA), (SPRY4) anti-Sprouty4, NRAS, BRAF (1:1000, Santa Cruz Biotechnology, Dallas, TX), and mouse monoclonal anti-GAPDH (1:40,000, Abcam, Cambridge, MA) for 2 h, and horseradish peroxidase-conjugated secondary antibody (1:4000) for 1 h. Antigen–antibody complexes were detected by ECL enhanced chemiluminescence solution (Bio-Rad, Hercules, CA). The signal intensity was quantified using ImageJ analysis software [31]. To clearly demonstrate the difference, the relative gray-scale value of target protein vs. GAPDH of the control group was set as 1. Results shown are representative of three independent experiments. Phospho-kinase screening was performed using a phospho-kinase array (R&D Systems, Inc., Minneapolis, MN, ARY003B) as per the manufacturer’s instructions. Briefly, as discussed above, whole cell lysates of 1 × 10^7^ cells/ml were subjected for phosphokinase array (Data not shown).

### Cell viability assay

To examine the growth retarding effect of dual oncogenes on the viability of doxycycline-induced melanoma cells, Tet-On BRAF(V600E) (SK-MEL-119^NRAS*^ + iBRAF*, SK-MEL-63^NRAS*^ + iBRAF* and WM-1361^NRAS*^ + iBRAF*) and Tet-OnNRAS(Q61R) (GMEL^BRAF*^ + iNRAS*, MGH-CH-1^BRAF*^ + iNRAS* and A375^BRAF*^ + iNRAS*) melanoma cell lines were seeded into 96-well white plates at a density of 1 × 10^3^ cells per well in 150 µl Tet-free growth media with or without doxycycline (100 ng/ml) and incubated for 6 days. A luminescence-based commercial kit (CellTiter-Glo; Promega) was used to measure cell viability. Briefly, 30 µl of cell lysis/ATP detection reagent was added to each well and incubated on a shaking platform for 15 min at room temperature, and the luminescence was measured with a plate reader (Molecular Devices). Cell viability was calculated as fold change using GraphPad Prism 6 software [28].

### Cellular colony formation assay

Melanoma cells were seeded in 12-well plates at a density of 50−200 cells per well. The media were changed every other day, and the colonies were counted at day 10–14th after staining with 0.1% of crystal violet for at least an hour as described previously [27]. The number of colonies was determined by counting entire field of view from triplicate wells for each cell line under an Olympus SZ-PT Stereoscope using a ×10 eyepiece. The data were expressed as means ± SD of at least three independent experiments.

### Cell cycle analysis

Cell cycle analyses were performed to evaluate the distribution of cells in various cell cycle phases (subG1, G1, S, and G2/M) by measuring the DNA content of nuclei labeled with propidium iodide (PI) (Life Technologies). Briefly, Tet-On BRAF(V600E) cells (SK-MEL-119^NRAS*^ + iBRAF*, WM1361^NRAS*^ + iBRAF*, and SK-MEL-63^NRAS*^ + iBRAF*) and Tet-On NRAS(Q61R) (GMEL^BRAF*^ + iNRAS* and A375^BRAF*^ + iNRAS*) viable cells were plated in triplicates at 2.0 × 10^5^ cells per well in six-well plates and incubated with and without doxycycline (0.1 µg/ml) for 4–5 days at 37 °C in humidified 5% CO_2_ incubator. After trypsinization cells were harvested and prepared single-cell suspension in 1 × PBS buffer. Cells were washed twice and centrifuged at 300 × *g* for 5 min, resuspended ~6 × 10^6^ cells/ml, 500 µl cell suspension were aliquoted into two polypropylene tubes one for cell cycle and another for apoptosis. For cell cycle, cells were fixed with ice-cold 70% (v/v) ethanol drop wise while gently vortexing and kept at −20 °C overnight prior to propidium iodide (PI) staining and flow cytometric analysis. Cells were centrifuged and washed twice with cold 1× PBS and added 0.5 ml of propidium iodide staining solution to cell pellet and mix well (100 µg/ml of propidium iodide and 100 µg/ml of RNase A in 0.1%Triton X-100) in 1× PBS (Sigma-Aldrich, St. Louis, MO) and incubated at room temperature in dark for 30 min [27]. Samples were analyzed by flow cytometry (BD FACS Calibur flow cytometer, BD Biosciences-US, Sparks Glencoe, MD). FlowJo, version 7.6.5 software (Ashland, OR) was used to calculate the percentages of cells in various cell cycle phases. All experiments were performed at least three times in triplicate.

### Apoptosis assay

Cells were washed with PBS, incubated with Annexin-V Alexa Fluor 647 and 4′,6-diamidino-2-phenylindole (DAPI) for 15 minutes at room temperature in the dark as per the manufacturer’s protocol (Life Technologies). The percentage of Annexin-V-positive cells was determined by flow cytometry BD FACSAria, (BD Biosciences-US) and results were analyzed using FlowJo, version 7.6.5 software (Ashland, OR). As stated before, the cell cycle and apoptosis assays were performed in parallel and in triplicate for each condition.

### Senescence assay

Cell senescence was measured by detection of SA-ß-gal activity using the Senescence Detection Kit (K#320-250, BioVision, Milpitas, CA) according to the manufacturer’s instruction. To prepare oncogene-induced secondary senescent melanoma cells for these assays, Tet-On BRAF(V600E) (SK-MEL-119^NRAS*^ + iBRAF*, WM1361^NRAS*^ + iBRAF*, and SK-MEL-63^NRAS*^ + iBRAF*) and Tet-On NRAS(Q61R) (GMEL^BRAF*^ + iNRAS*, and A375^BRAF*^ + iNRAS*) viable melanoma cells were induced with or without doxycycline (50–100 ng/ml) for 3 days. The cells were then subjected to senescence-associated expression of ß-galactosidase (SA-ß-gal assay) activity as per the manufacturer’s instruction (BioVision, Milpitas, CA).

### Q-PCR analysis

Cells were seeded in 10 cm plates at a density of 1.0×10^6^ cells/well and allowed to adhere overnight in Tet-free growth medium. Next day, medium was replaced with or without doxycycline 50–100 ng/ml for a defined time of 4–5 days before total RNA was isolated using RNeasy isolation kit (Qiagen, Germany) according to the manufacturer’s instructions. The first strand cDNA was reverse transcribed from 2 µg of RNA using high-capacity RNA to cDNA kit according to the manufacturer’s instructions (Applied Biosystems, Foster City, CA, USA). Microarray data were verified by qPCR using the gene-specific primers (Invitrogen; *SPRY4* (Hs01935412_s1), *NRAS* (Hs 00180035-m1), *BRAF* (Hs 00269944-m1), and: Hu-*GUSB*-FAM-4333767F), IDT: h*MITF*-M27 and TaqMan Master Mix (Roche Diagnostics Cor. Indianapolis, IN, USA). The relative expression of target genes vs. a reference gene, Hu-*GUSB*-FAM, was calculated using Ct values/or a built-in mathematical model (Roche Molecular Diagnostics, Branchburg, NJ) which included an efficiency correction for real-time PCR.

### Microarray preparation and analysis

Cells were seeded 24 h before treatment with or without doxycycline 50–100 ng/ml for a defined time of 4–5 days. RNA was extracted from 2 × 10^6^ cells from each condition (biologic duplicates) using the RNeasy Mini Kit (Qiagen). The RNA specimens were submitted to the Massachusetts Institute of Technology (MIT) Core Facility and profiled with Affymetrix Primeview GeneChip arrays as per the standard operating procedures of the Core Facility. Probe set intensity values were converted into log2 space after adding a pseudo-count of 1. Expressed genes were those with log2(expression) > log2(100 units), which is approximately log2 ∼6.64. Only transcript probes which were expressed in at least three or more samples were used for the comparative analysis. The effect of BRAF* or NRAS* was quantified as log2-fold differences using the formula: 2 Oncogene effectlog_2-fold_ = [Oncogene(+Dox) log_2-expression_ − Oncogene(−Dox) log_2-expression_] − [Vector(+Dox) log_2-expression_ − Vector(-Dox) log_2-expression_]. The entire expression set in log2 expression levels is shown in Table S1.

### Functional enrichment analysis using DAVID

Probes that exhibited >2-fold change (+1.0 or −1.0 log2-fold) for each line (Tables S2-S4) and for probes that were shared in SK-MEL-119^NRAS*^ + iBRAF* and GMEL^BRAF*^ + iNRAS* but NOT A375^BRAF*^ + iNRAS* (Table S5) were subjected to DAVIDV6.8 (https://david.ncifcrf.gov/) functional clustering annotation under the default mode. Per DAVID’s website for functional clustering, “The geometric mean (in -log scale) of member's *p* values in a corresponding annotation cluster, is used to rank their biological significance. Thus, the top ranked annotation groups most likely have consistent lower *p* values for their annotation members.” Annotation clustering Enrichment Scores are presented in Tables S2–S5. The annotation cluster is designated by the first GO term within the cluster, which by convention, has the lowest *p* value in the cluster.

### In vivo tumor growth assay

Sixteen Nod-SCID-gamma male mice of 6-week-old (Jackson Laboratory, Bar Harbor, ME) were divided into two groups, eight mice per group were injected subcutaneously (s.c.) with 0.2 million SK-MEL-119^NRASQ61R^ cells constitutively expressing either control CD516B2-vector or CD516B2-SPRY4 in 0.1 ml 10% DMEM growth medium per flank of mice. Animals were monitored twice weekly for 7 weeks. Body weights and tumor size were measured every week as described previously [27]. Data were expressed as mean ± SD, *N* = 8. Tumor histology was confirmed by hematoxylin/eosin staining of formalin-fixed and paraffin-embedded tissue. Animals were maintained in well-ventilated animal facility and tested in accordance with the MGH Animal Care and Use Committee guidelines.

### Immunohistochemistry, H&E and TUNEL

Immunohistochemistry, phosphate-buffered formalin fixed tumor tissues were paraffin-embedded and 5-µM-thick sections were collected onto poly-l-lysine-coated slides. The assays were performed as described previously [32]. Briefly, the sections were subjected for IHC, H&E and TUNEL. Two archival tumor sections per slide were deparaffinized at 60 °C for 1 h followed by a few CitriSolv dips and gradually rehydrated through graded ethanol. (1) IHC, Antigen retrieval (1× DAKO, Cat# S1699) was performed at 98 °C water bath for 30 min, permeabilization in 0.1% TritonX-100 TBS at 25 °C for 15 min. Endogenous AP and peroxidase blocking in 3% H_2_O_2_ solution for 15 min at 25 °C followed by protein blocking serum (10% goat serum, 3% BSA, 0.1% Tween-20 in TBS Sigma-Aldrich, Natick, MA) for 30 min. After this, tissue sections were incubated for 1 h, with primary antibodies such as anti-Ki67 for proliferation, anti-sprouty4 for overexpression, ß-galactosidase for senescence, followed by a secondary alkaline phosphatase antibody, either MACH2 mouse AP-polymer detection (BioCare Medical, Cat# MALP521-G) or MACH 2 rabbit AP-polymer detection (BioCare Medical, Cat# RALP525-G) for 30 min. The slides were developed with Vulcan Fast Red Chromogen kit2 (BioCare Medical, Cat# FR805 H) and counter stained with Mossberg hematoxylin (Vector H-3401). The colorimetric signals were detected with digital slide scanner NanoZoomer-2.0HT and analyzed by NDP.view2 software (Hamamatsu Photonics K.K., Hamamatsu, Japan). (2) H&E, after rehydration hematoxylin and eosin staining was carried out using the Mossberg labs protocol. (3) TUNEL staining was performed using Dead End Fluorometric TUNEL kit (Promega) according to the manufacturer’s instruction.

### Cell line authentication

All cell lines were stratified to three levels of confidence after STR genotyping and MITF expression analysis. Those lines that matched the STR genotyping database were considered “CONFIRMED” as the designated line. Cell lines with no STR hits but had mutational data at melanoma driver loci (e.g. BRAF, NRAS, PTEN, etc.) were manually compared to public datasets such as COSMIC or individually referenced papers. If the mutational profile of the cell line matched independent public data (i.e. results not from our laboratory), these lines were labeled “CONSISTENT” with the designated line. Lastly, cell lines that were either newly developed or lacked STR information and public domain information were experimentally assayed for MITF levels by RNAseq. Those that showed MITF expression were considered “COMPATIBLE” with melanoma. The following represents the level of confirmation for cell lines in this study: A375^BRAF*^ (CONFIRMED), GMEL^BRAF*^ (CONFIRMED), WM1361^NRAS*^ (CONFIRMED), SK-MEL-63^NRAS*^ (CONSISTENT) and MGH-CH-1^BRAF*^ (COMPATIBLE). The cell lines were thawed and collected between third or fourth passages for original naive and stably expressing transductant cells. After experiment, the cell lines were either used up or discarded at 15th to 25th day. All used cell lines were mycoplasma tested as instructed by the company (Invitrogen).

### Statistical analysis

Data from different experiments were represented as means ± SD from at least three independent experiments. To analyze cell viability, linear regression analysis was performed using GraphPad Prism 7.0 (GraphPad Software, La Jolla, CA). Significance was established at *p* = 0.05, as usual.

### Animal material

The mice experiments were performed in accordance with a protocol approved by the Institutional Animal Care and Use Committee (IACUC) of MGH.

## Supplementary information

Supplementary fig 1

supplementary fig 2

supplementary figure 3

supplementary figure 4

supplementary figure 5

supplementary figure 6

supplementary figure 7

supplementary figure 8

supplementary figure 9

supplementary figure 10

supplementary figure 11

Materials and Methods-SUPPLEMENTARY

Kumar et al, NRAS BRAF Oncogene TABLES SUPPLEMENTARY
