# Supplementary material for: Growth suppression by dual BRAF(V600E) and NRAS(Q61) oncogene expression is mediated by SPRY4 in melanoma
Source: Oncogene. 2019 Jan 16;38(18):3504–20. doi: 10.1038/s41388-018-0632-2 (PMC6756020; doi:10.1038/s41388-018-0632-2)
Supplement: Supplementary file 1 — Supplementary fig 1 [file 41388_2018_632_MOESM1_ESM.pptx]

## Slide 1
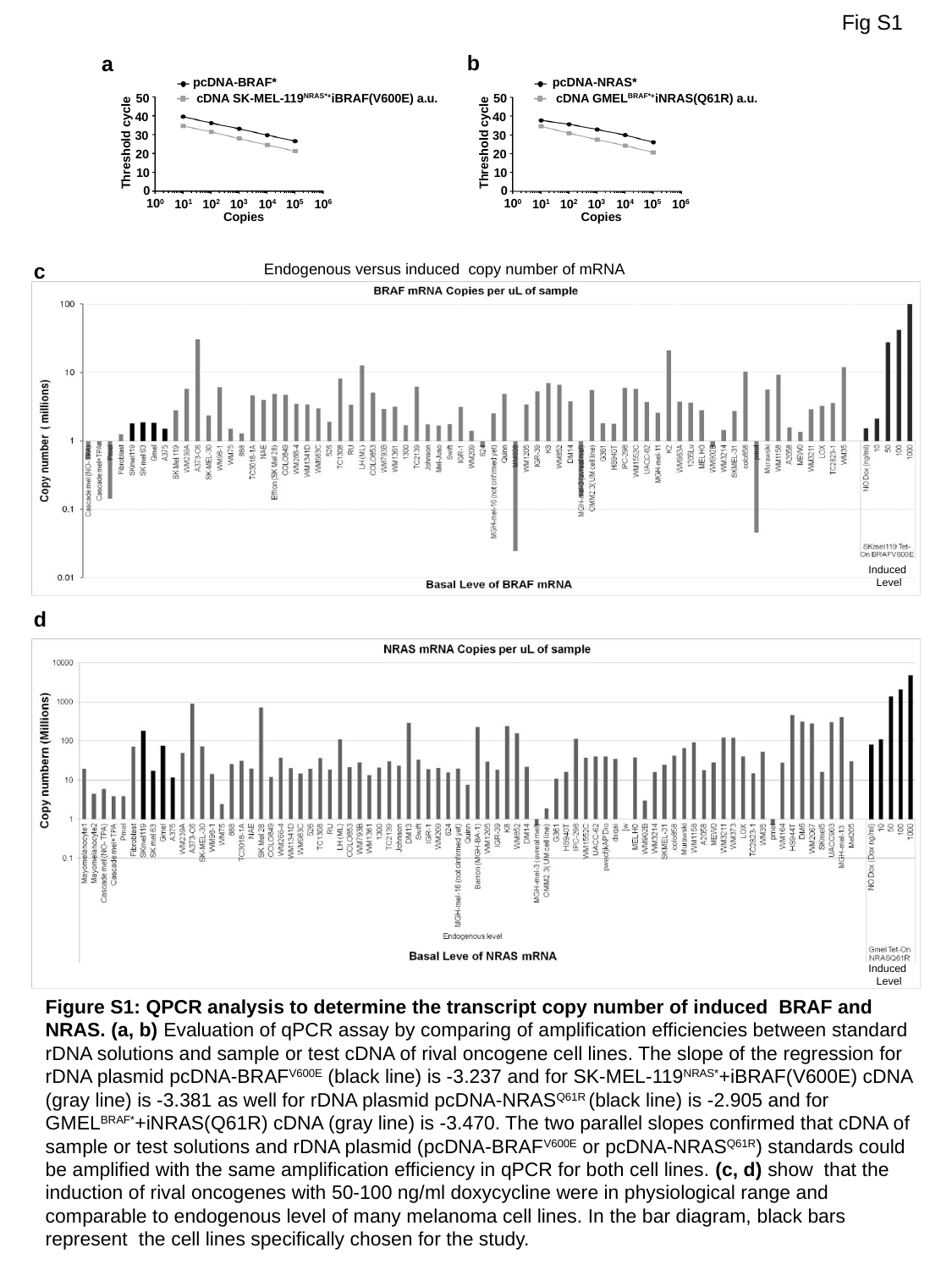

Fig S1
b
a
pcDNA-NRAS*
cDNA GMELBRAF*+iNRAS(Q61R) a.u.
50
40
30
Threshold cycle
20
10
0
100
101
102
105
106
103
104
Copies
pcDNA-BRAF*
cDNA SK-MEL-119NRAS*+iBRAF(V600E) a.u.
50
40
30
Threshold cycle
20
10
0
100
101
102
105
106
103
104
Copies
c
Endogenous versus induced copy number of mRNA
Induced
Level
d
Induced
Level
Figure S1: QPCR analysis to determine the transcript copy number of induced BRAF and NRAS. (a, b) Evaluation of qPCR assay by comparing of amplification efficiencies between standard rDNA solutions and sample or test cDNA of rival oncogene cell lines. The slope of the regression for rDNA plasmid pcDNA-BRAFV600E (black line) is -3.237 and for SK-MEL-119NRAS*+iBRAF(V600E) cDNA (gray line) is -3.381 as well for rDNA plasmid pcDNA-NRASQ61R (black line) is -2.905 and for GMELBRAF*+iNRAS(Q61R) cDNA (gray line) is -3.470. The two parallel slopes confirmed that cDNA of sample or test solutions and rDNA plasmid (pcDNA-BRAFV600E or pcDNA-NRASQ61R) standards could be amplified with the same amplification efficiency in qPCR for both cell lines. (c, d) show that the induction of rival oncogenes with 50-100 ng/ml doxycycline were in physiological range and comparable to endogenous level of many melanoma cell lines. In the bar diagram, black bars represent the cell lines specifically chosen for the study.
