# Supplementary material for: Growth suppression by dual BRAF(V600E) and NRAS(Q61) oncogene expression is mediated by SPRY4 in melanoma
Source: Oncogene. 2019 Jan 16;38(18):3504–20. doi: 10.1038/s41388-018-0632-2 (PMC6756020; doi:10.1038/s41388-018-0632-2)
Supplement: Supplementary file 2 — supplementary fig 2 [file 41388_2018_632_MOESM2_ESM.pptx]

## Slide 1
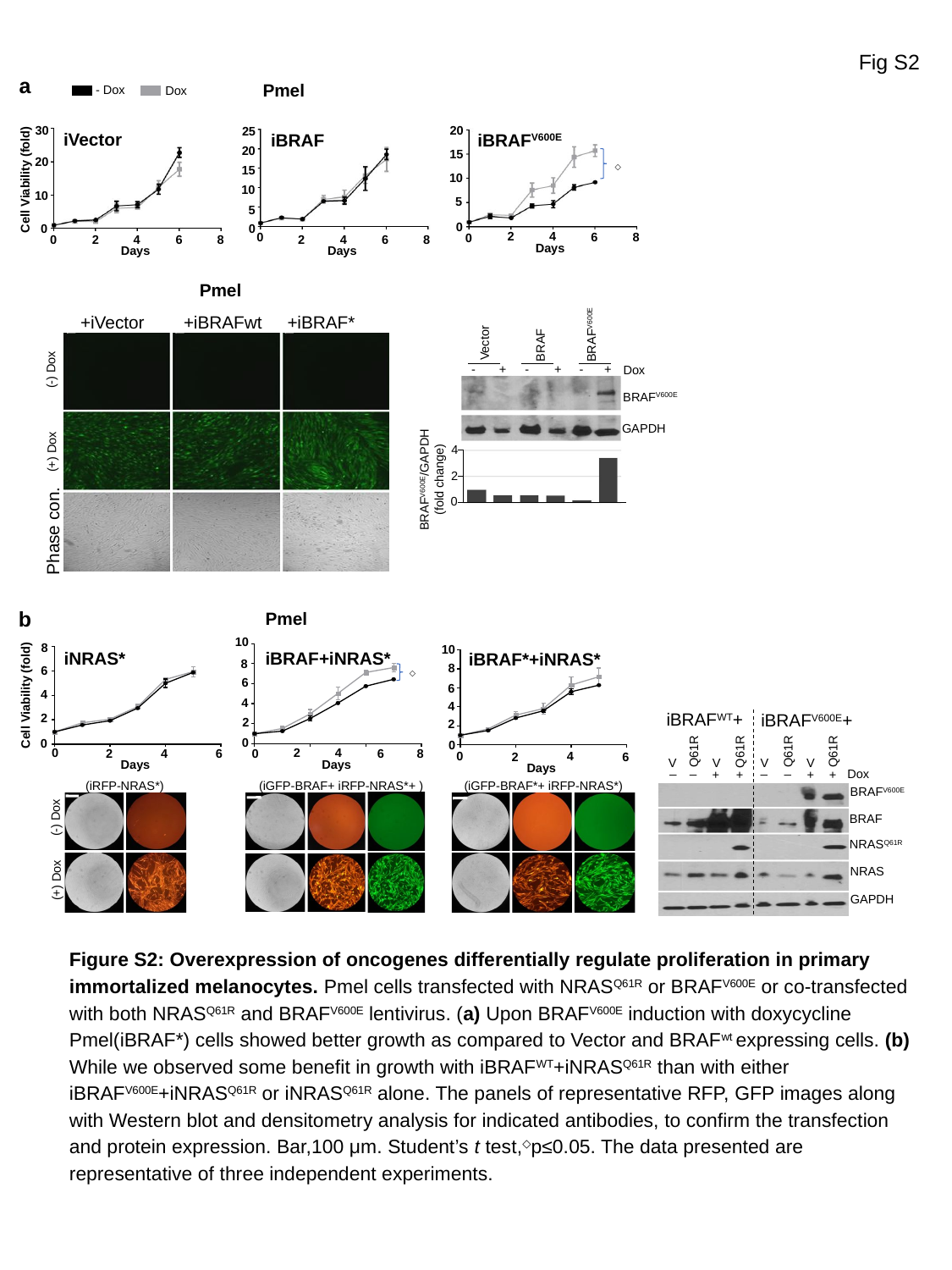

Fig S2
a
Pmel
 - Dox
Dox
30
20
10
0
8
6
4
2
0
Days
20
15
10
5
0
4
2
6
8
0
Days
iBRAFV600E
BRAFV600E
25
20
15
10
5
0
0
8
6
4
2
Days
iVector
iBRAF
◇
Cell Viability (fold)
BRAFV600E
Vector
BRAF
-
+
-
+
-
+
Dox
BRAFV600E
GAPDH
Pmel
+iVector
+iBRAFwt
+iBRAF*
Phase con.
(-) Dox
4
 (+) Dox
BRAFV600E/GAPDH (fold change)
2
0
b
Pmel
10
8
6
4
2
0
4
2
0
8
6
Days
8
6
4
2
0
0
6
4
2
Days
10
8
6
4
2
0
4
0
6
2
Days
iBRAF+iNRAS*
iNRAS*
iBRAF*+iNRAS*
◇
Cell Viability (fold)
iBRAFWT+
iBRAFV600E+
Q61R
Q61R
Q61R
Q61R
V
V
V
V
_
_
_
_
Dox
+
+
+
+
BRAF
NRAS
GAPDH
(iGFP-BRAF+ iRFP-NRAS*+ )
(iRFP-NRAS*)
(iGFP-BRAF*+ iRFP-NRAS*)
BRAFV600E
(-) Dox
NRASQ61R
 (+) Dox
Figure S2: Overexpression of oncogenes differentially regulate proliferation in primary immortalized melanocytes. Pmel cells transfected with NRASQ61R or BRAFV600E or co-transfected with both NRASQ61R and BRAFV600E lentivirus. (a) Upon BRAFV600E induction with doxycycline Pmel(iBRAF*) cells showed better growth as compared to Vector and BRAFwt expressing cells. (b) While we observed some benefit in growth with iBRAFWT+iNRASQ61R than with either iBRAFV600E+iNRASQ61R or iNRASQ61R alone. The panels of representative RFP, GFP images along with Western blot and densitometry analysis for indicated antibodies, to confirm the transfection and protein expression. Bar,100 μm. Student’s t test,◇p≤0.05. The data presented are representative of three independent experiments.
