# Supplementary material for: Growth suppression by dual BRAF(V600E) and NRAS(Q61) oncogene expression is mediated by SPRY4 in melanoma
Source: Oncogene. 2019 Jan 16;38(18):3504–20. doi: 10.1038/s41388-018-0632-2 (PMC6756020; doi:10.1038/s41388-018-0632-2)
Supplement: Supplementary file 4 — supplementary figure 4 [file 41388_2018_632_MOESM4_ESM.pptx]

## Slide 1
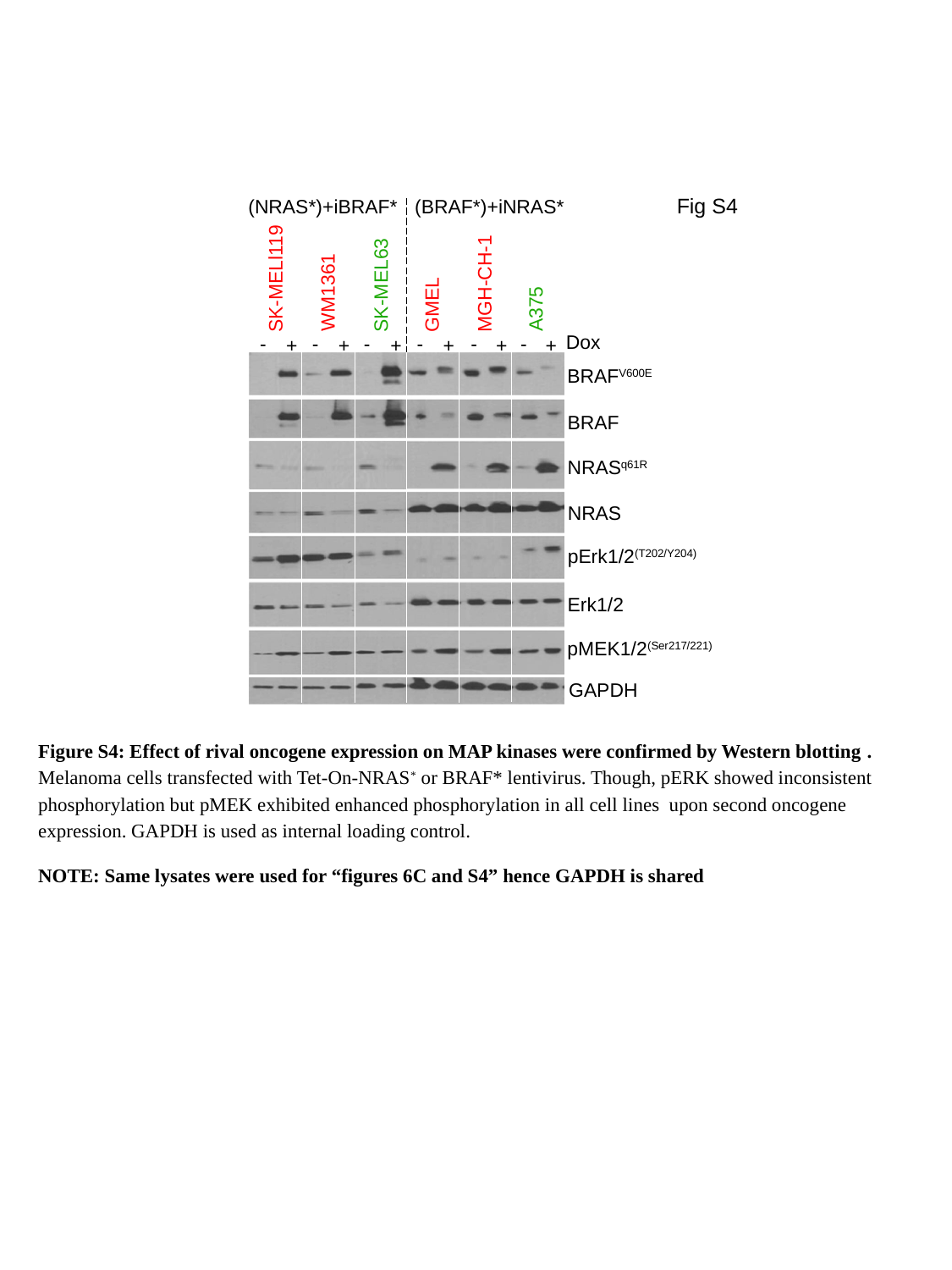

Fig S4
(BRAF*)+iNRAS*
(NRAS*)+iBRAF*
SK-MELl119
MGH-CH-1
SK-MEL63
GMEL
WM1361
A375
Dox
-
-
-
-
-
-
+
+
+
+
+
+
BRAFV600E
BRAF
NRASq61R
NRAS
pErk1/2(T202/Y204)
Erk1/2
pMEK1/2(Ser217/221)
GAPDH
Figure S4: Effect of rival oncogene expression on MAP kinases were confirmed by Western blotting . Melanoma cells transfected with Tet-On-NRAS* or BRAF* lentivirus. Though, pERK showed inconsistent phosphorylation but pMEK exhibited enhanced phosphorylation in all cell lines upon second oncogene expression. GAPDH is used as internal loading control.
NOTE: Same lysates were used for “figures 6C and S4” hence GAPDH is shared
