# Supplementary material for: Growth suppression by dual BRAF(V600E) and NRAS(Q61) oncogene expression is mediated by SPRY4 in melanoma
Source: Oncogene. 2019 Jan 16;38(18):3504–20. doi: 10.1038/s41388-018-0632-2 (PMC6756020; doi:10.1038/s41388-018-0632-2)
Supplement: Supplementary file 5 — supplementary figure 5 [file 41388_2018_632_MOESM5_ESM.pptx]

## Slide 1
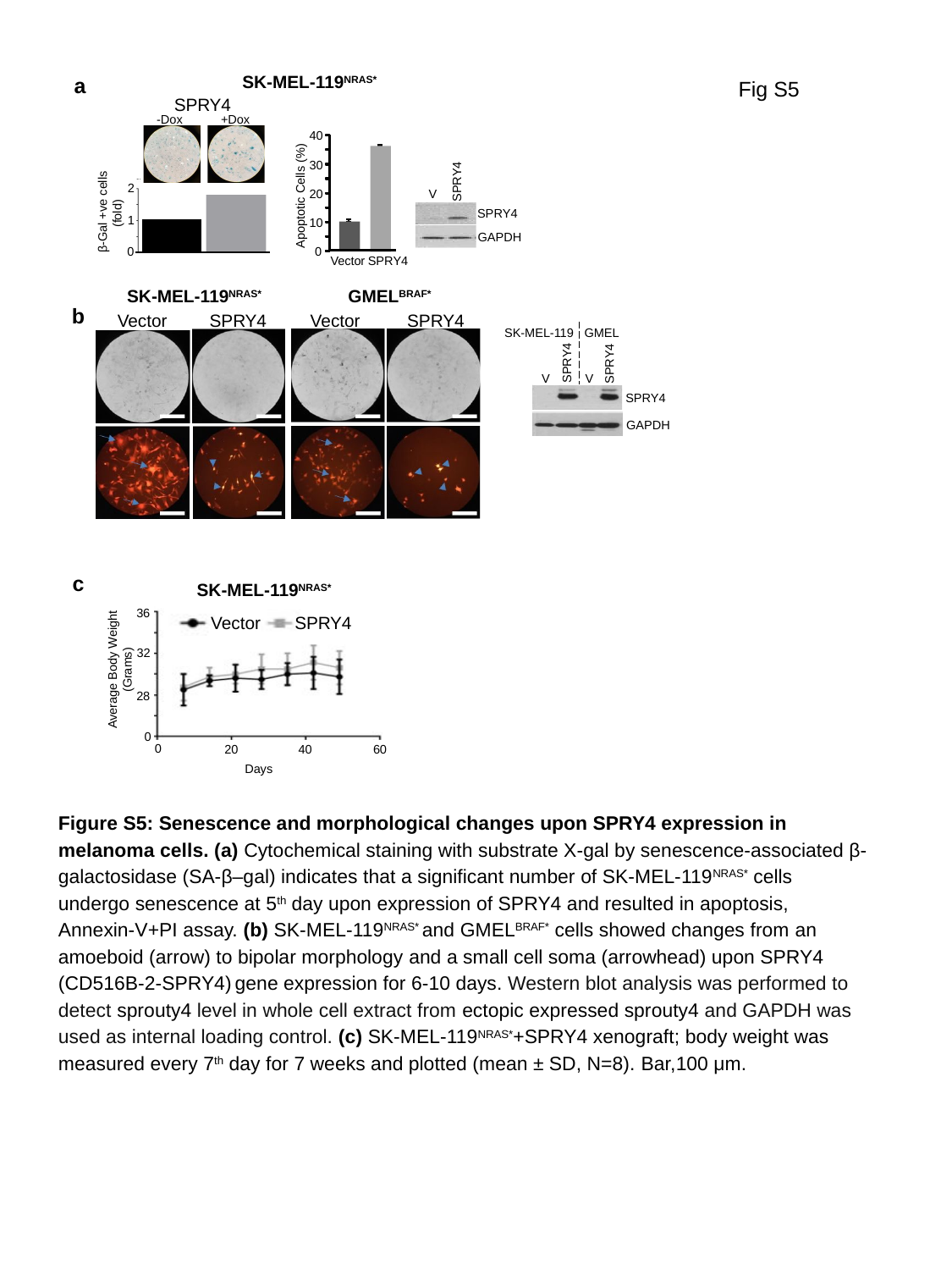

SK-MEL-119NRAS*
a
Fig S5
SPRY4
-Dox
+Dox
2
1
0
 β-Gal +ve cells
(fold)
40
SPRY4
V
SPRY4
GAPDH
30
Apoptotic Cells (%)
20
10
0
Vector
SPRY4
SK-MEL-119NRAS*
GMELBRAF*
b
Vector
SPRY4
Vector
SPRY4
SK-MEL-119
GMEL
SPRY4
SPRY4
V
V
SPRY4
GAPDH
c
SK-MEL-119NRAS*
36
SPRY4
Vector
32
Average Body Weight (Grams)
28
0
0
20
40
60
Days
Figure S5: Senescence and morphological changes upon SPRY4 expression in melanoma cells. (a) Cytochemical staining with substrate X-gal by senescence-associated β-galactosidase (SA-β–gal) indicates that a significant number of SK-MEL-119NRAS* cells undergo senescence at 5th day upon expression of SPRY4 and resulted in apoptosis, Annexin-V+PI assay. (b) SK-MEL-119NRAS* and GMELBRAF* cells showed changes from an amoeboid (arrow) to bipolar morphology and a small cell soma (arrowhead) upon SPRY4 (CD516B-2-SPRY4) gene expression for 6-10 days. Western blot analysis was performed to detect sprouty4 level in whole cell extract from ectopic expressed sprouty4 and GAPDH was used as internal loading control. (c) SK-MEL-119NRAS*+SPRY4 xenograft; body weight was measured every 7th day for 7 weeks and plotted (mean ± SD, N=8). Bar,100 μm.
