# Supplementary material for: Growth suppression by dual BRAF(V600E) and NRAS(Q61) oncogene expression is mediated by SPRY4 in melanoma
Source: Oncogene. 2019 Jan 16;38(18):3504–20. doi: 10.1038/s41388-018-0632-2 (PMC6756020; doi:10.1038/s41388-018-0632-2)
Supplement: Supplementary file 6 — supplementary figure 6 [file 41388_2018_632_MOESM6_ESM.pptx]

## Slide 1
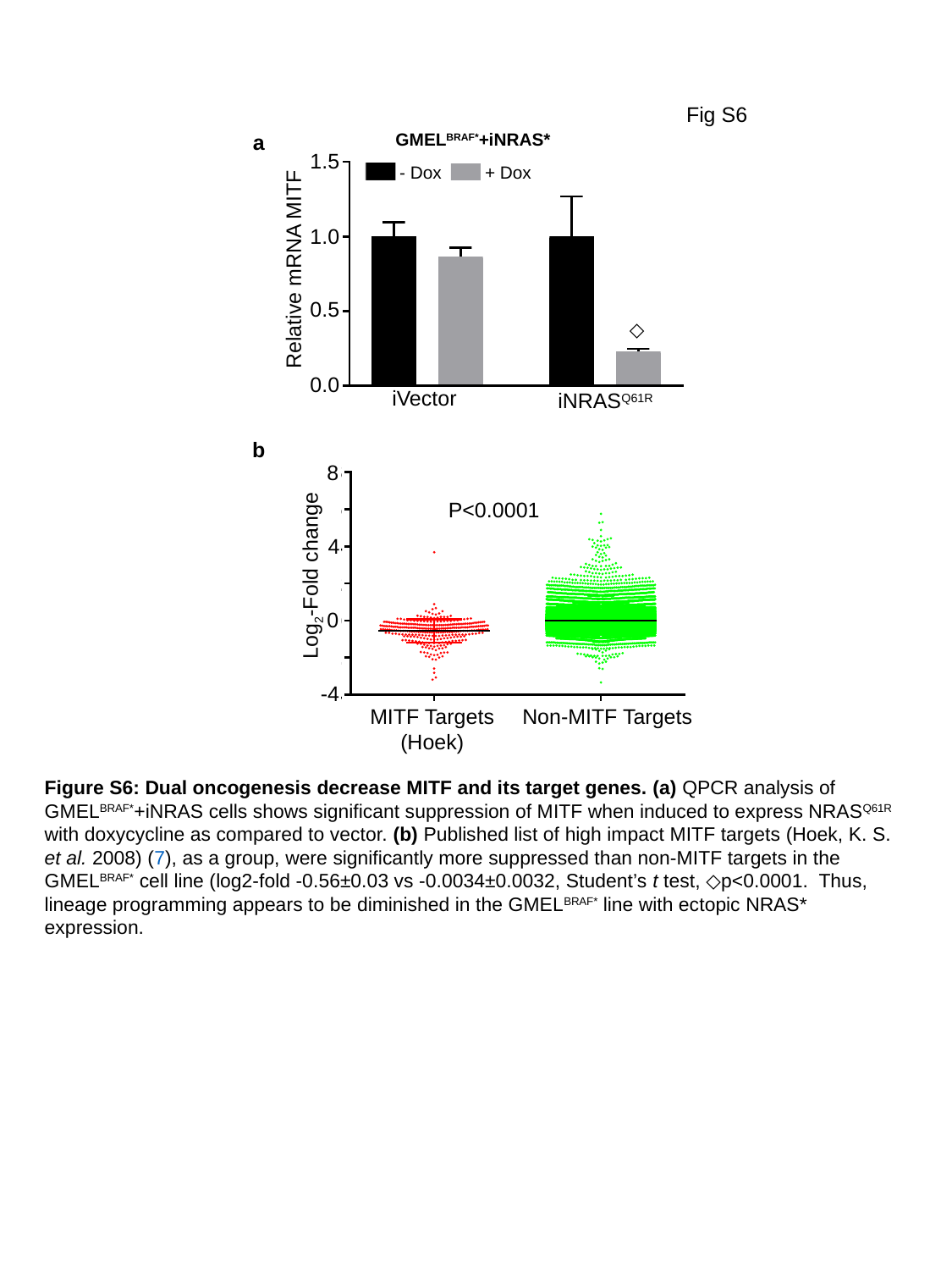

Fig S6
GMELBRAF*+iNRAS*
a
1.5
- Dox
+ Dox
1.0
Relative mRNA MITF
0.5
◇
0.0
iVector
iNRASQ61R
b
8
P<0.0001
4
Log2-Fold change
0
-4
MITF Targets
(Hoek)
Non-MITF Targets
Figure S6: Dual oncogenesis decrease MITF and its target genes. (a) QPCR analysis of GMELBRAF*+iNRAS cells shows significant suppression of MITF when induced to express NRASQ61R with doxycycline as compared to vector. (b) Published list of high impact MITF targets (Hoek, K. S. et al. 2008) (7), as a group, were significantly more suppressed than non-MITF targets in the GMELBRAF* cell line (log2-fold -0.56±0.03 vs -0.0034±0.0032, Student’s t test, ◇p<0.0001. Thus, lineage programming appears to be diminished in the GMELBRAF* line with ectopic NRAS* expression.
