# Supplementary material for: Growth suppression by dual BRAF(V600E) and NRAS(Q61) oncogene expression is mediated by SPRY4 in melanoma
Source: Oncogene. 2019 Jan 16;38(18):3504–20. doi: 10.1038/s41388-018-0632-2 (PMC6756020; doi:10.1038/s41388-018-0632-2)
Supplement: Supplementary file 7 — supplementary figure 7 [file 41388_2018_632_MOESM7_ESM.pptx]

## Slide 1
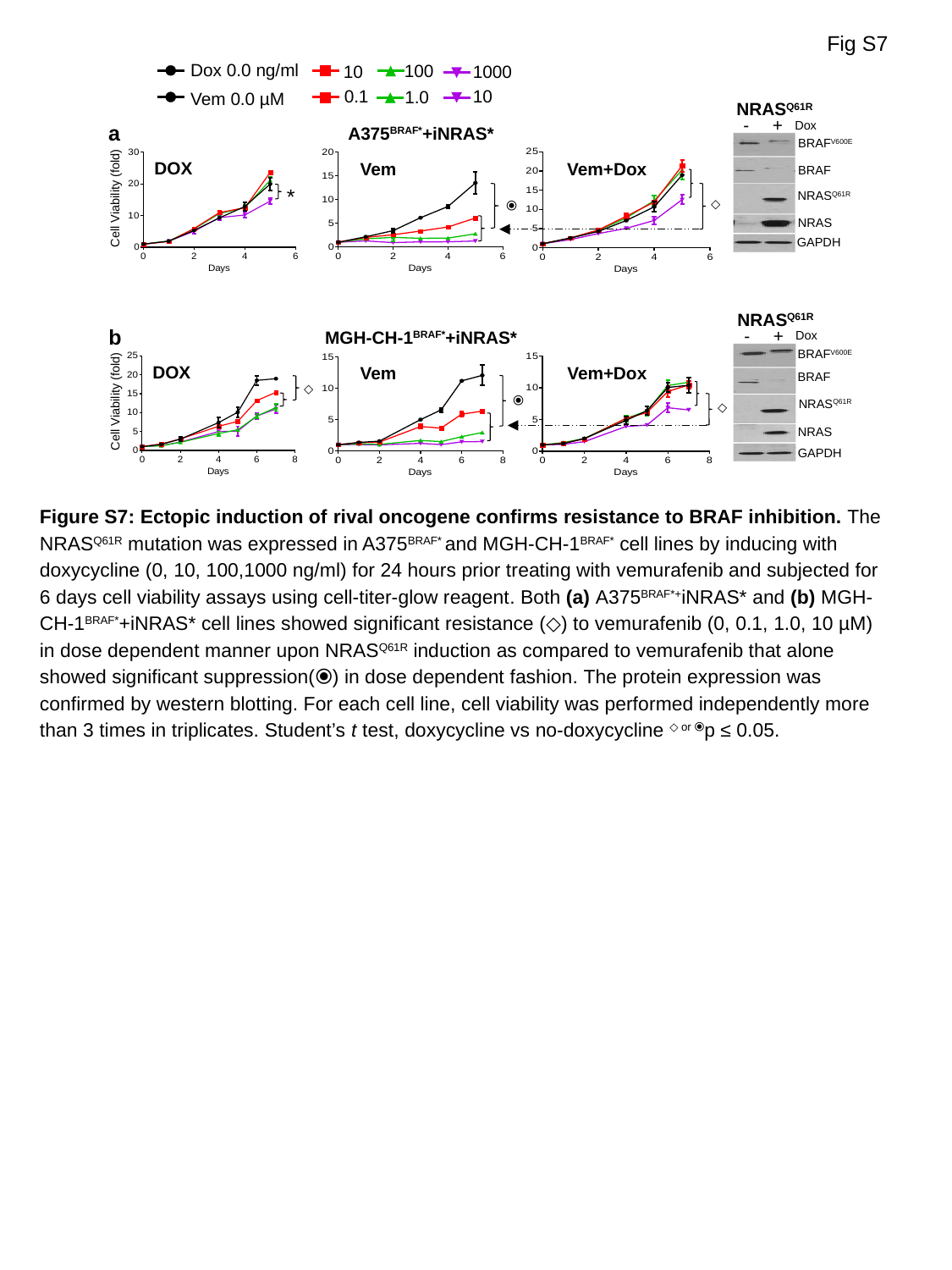

Fig S7
Dox 0.0 ng/ml
100
10
1000
10
0.1
 1.0
Vem 0.0 µM
NRASQ61R
-
+
Dox
BRAFV600E
BRAF
NRASQ61R
NRAS
GAPDH
a
A375BRAF*+iNRAS*
DOX
Vem
Vem+Dox
◇
*
⦿
Cell Viability (fold)
NRASQ61R
-
+
Dox
BRAFV600E
BRAF
NRASQ61R
NRAS
GAPDH
b
MGH-CH-1BRAF*+iNRAS*
DOX
Vem
Vem+Dox
◇
⦿
◇
Cell Viability (fold)
Figure S7: Ectopic induction of rival oncogene confirms resistance to BRAF inhibition. The NRASQ61R mutation was expressed in A375BRAF* and MGH-CH-1BRAF* cell lines by inducing with doxycycline (0, 10, 100,1000 ng/ml) for 24 hours prior treating with vemurafenib and subjected for 6 days cell viability assays using cell-titer-glow reagent. Both (a) A375BRAF*+iNRAS* and (b) MGH-CH-1BRAF*+iNRAS* cell lines showed significant resistance (◇) to vemurafenib (0, 0.1, 1.0, 10 µM) in dose dependent manner upon NRASQ61R induction as compared to vemurafenib that alone showed significant suppression(⦿) in dose dependent fashion. The protein expression was confirmed by western blotting. For each cell line, cell viability was performed independently more than 3 times in triplicates. Student’s t test, doxycycline vs no-doxycycline ◇ or ⦿p ≤ 0.05.
