# Supplementary material for: Growth suppression by dual BRAF(V600E) and NRAS(Q61) oncogene expression is mediated by SPRY4 in melanoma
Source: Oncogene. 2019 Jan 16;38(18):3504–20. doi: 10.1038/s41388-018-0632-2 (PMC6756020; doi:10.1038/s41388-018-0632-2)
Supplement: Supplementary file 8 — supplementary figure 8 [file 41388_2018_632_MOESM8_ESM.pptx]

## Slide 1
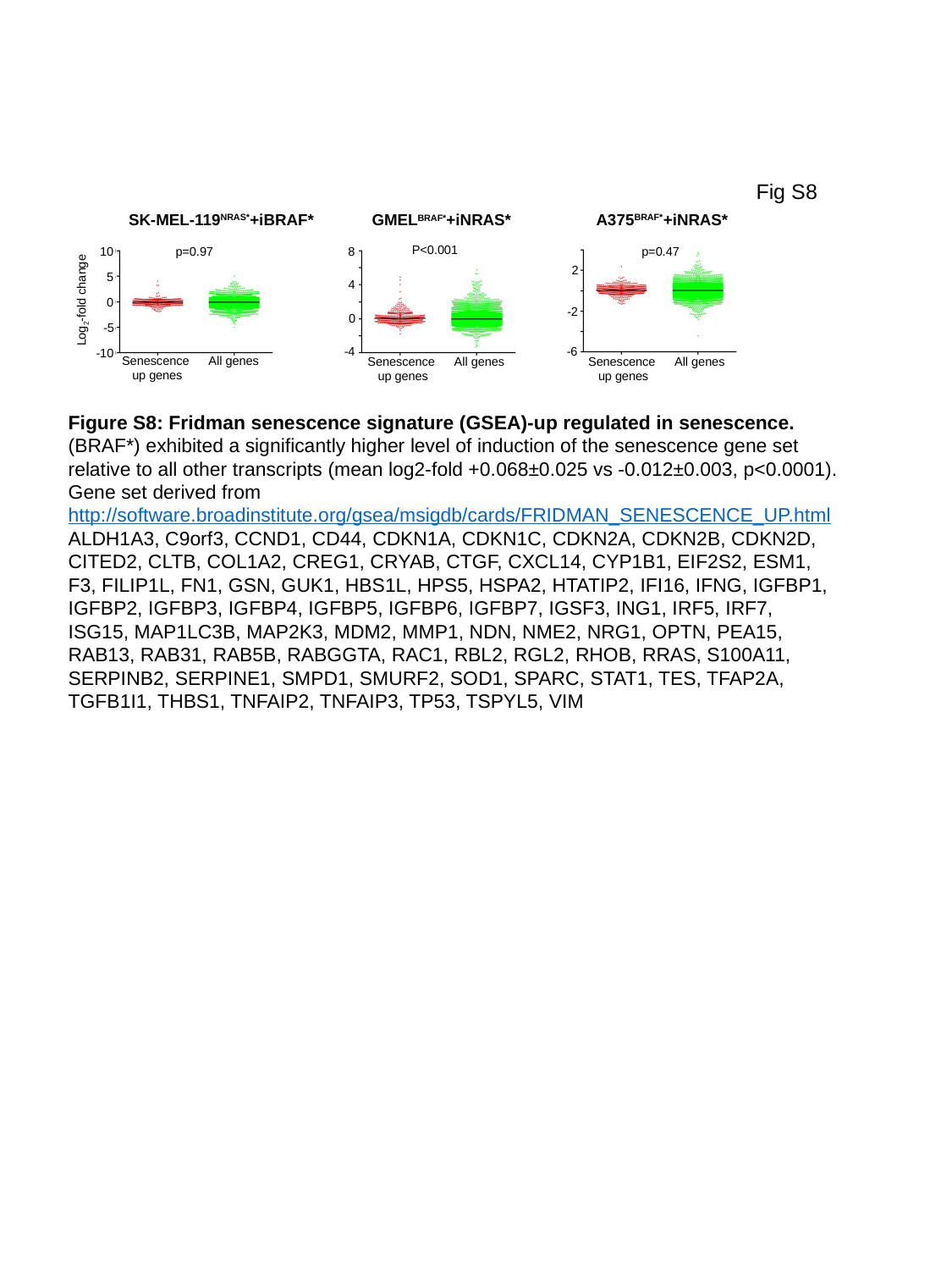

Fig S8
SK-MEL-119NRAS*+iBRAF*
A375BRAF*+iNRAS*
GMELBRAF*+iNRAS*
P<0.001
10
8
p=0.47
p=0.97
2
5
4
Log2-fold change
0
-2
0
-5
-6
-4
-10
All genes
Senescence
up genes
All genes
All genes
Senescence
up genes
Senescence
up genes
Figure S8: Fridman senescence signature (GSEA)-up regulated in senescence. (BRAF*) exhibited a significantly higher level of induction of the senescence gene set relative to all other transcripts (mean log2-fold +0.068±0.025 vs -0.012±0.003, p<0.0001). Gene set derived from http://software.broadinstitute.org/gsea/msigdb/cards/FRIDMAN_SENESCENCE_UP.html
ALDH1A3, C9orf3, CCND1, CD44, CDKN1A, CDKN1C, CDKN2A, CDKN2B, CDKN2D, CITED2, CLTB, COL1A2, CREG1, CRYAB, CTGF, CXCL14, CYP1B1, EIF2S2, ESM1, F3, FILIP1L, FN1, GSN, GUK1, HBS1L, HPS5, HSPA2, HTATIP2, IFI16, IFNG, IGFBP1, IGFBP2, IGFBP3, IGFBP4, IGFBP5, IGFBP6, IGFBP7, IGSF3, ING1, IRF5, IRF7, ISG15, MAP1LC3B, MAP2K3, MDM2, MMP1, NDN, NME2, NRG1, OPTN, PEA15, RAB13, RAB31, RAB5B, RABGGTA, RAC1, RBL2, RGL2, RHOB, RRAS, S100A11, SERPINB2, SERPINE1, SMPD1, SMURF2, SOD1, SPARC, STAT1, TES, TFAP2A, TGFB1I1, THBS1, TNFAIP2, TNFAIP3, TP53, TSPYL5, VIM
