# Supplementary material for: Growth suppression by dual BRAF(V600E) and NRAS(Q61) oncogene expression is mediated by SPRY4 in melanoma
Source: Oncogene. 2019 Jan 16;38(18):3504–20. doi: 10.1038/s41388-018-0632-2 (PMC6756020; doi:10.1038/s41388-018-0632-2)
Supplement: Supplementary file 9 — supplementary figure 9 [file 41388_2018_632_MOESM9_ESM.pptx]

## Slide 1
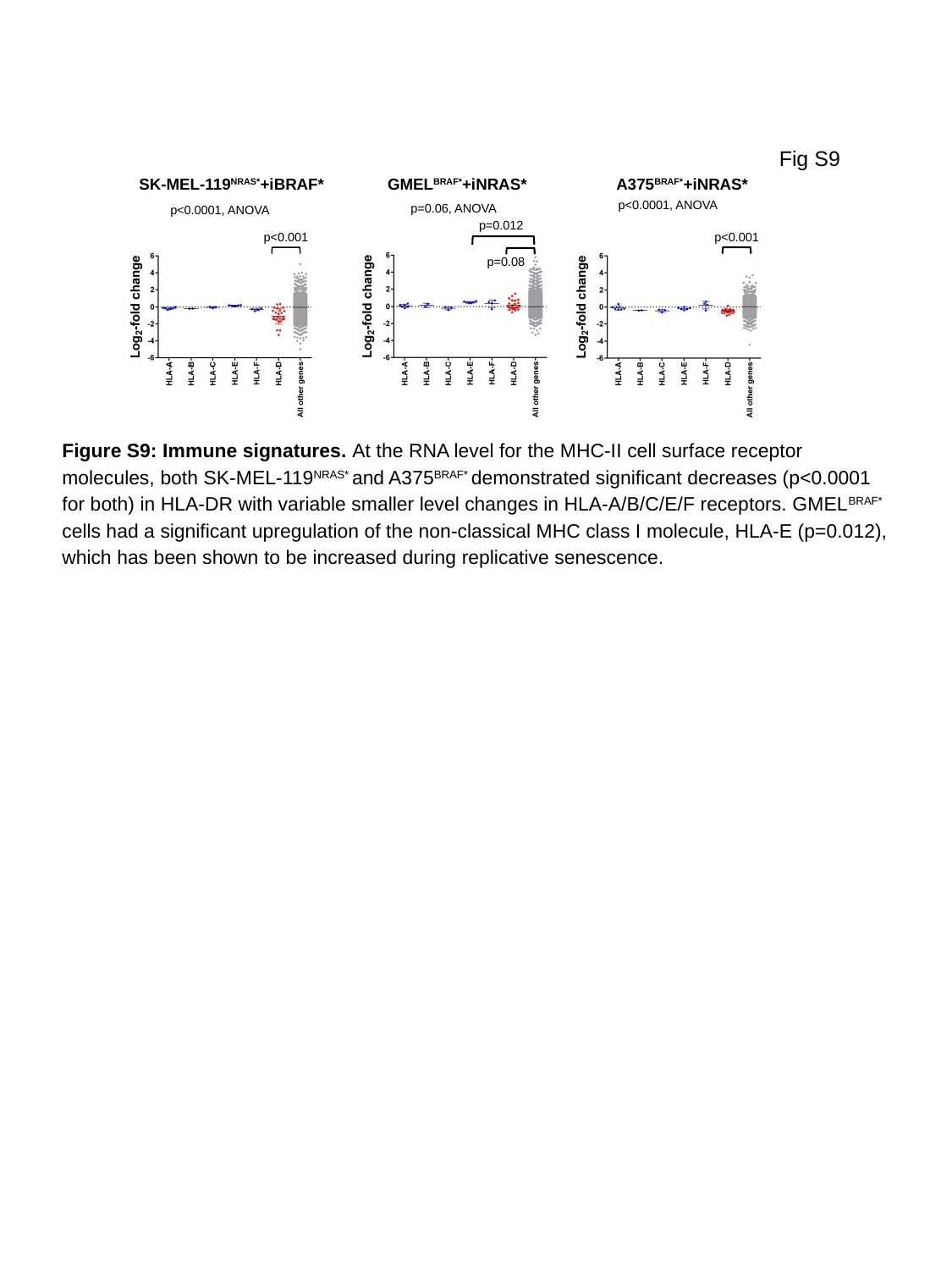

Fig S9
SK-MEL-119NRAS*+iBRAF*
GMELBRAF*+iNRAS*
A375BRAF*+iNRAS*
p<0.0001, ANOVA
p=0.06, ANOVA
p<0.0001, ANOVA
p=0.012
p=0.08
p<0.001
p<0.001
Figure S9: Immune signatures. At the RNA level for the MHC-II cell surface receptor molecules, both SK-MEL-119NRAS* and A375BRAF* demonstrated significant decreases (p<0.0001 for both) in HLA-DR with variable smaller level changes in HLA-A/B/C/E/F receptors. GMELBRAF* cells had a significant upregulation of the non-classical MHC class I molecule, HLA-E (p=0.012), which has been shown to be increased during replicative senescence.
