# Supplementary material for: Growth suppression by dual BRAF(V600E) and NRAS(Q61) oncogene expression is mediated by SPRY4 in melanoma
Source: Oncogene. 2019 Jan 16;38(18):3504–20. doi: 10.1038/s41388-018-0632-2 (PMC6756020; doi:10.1038/s41388-018-0632-2)
Supplement: Supplementary file 10 — supplementary figure 10 [file 41388_2018_632_MOESM10_ESM.pptx]

## Slide 1
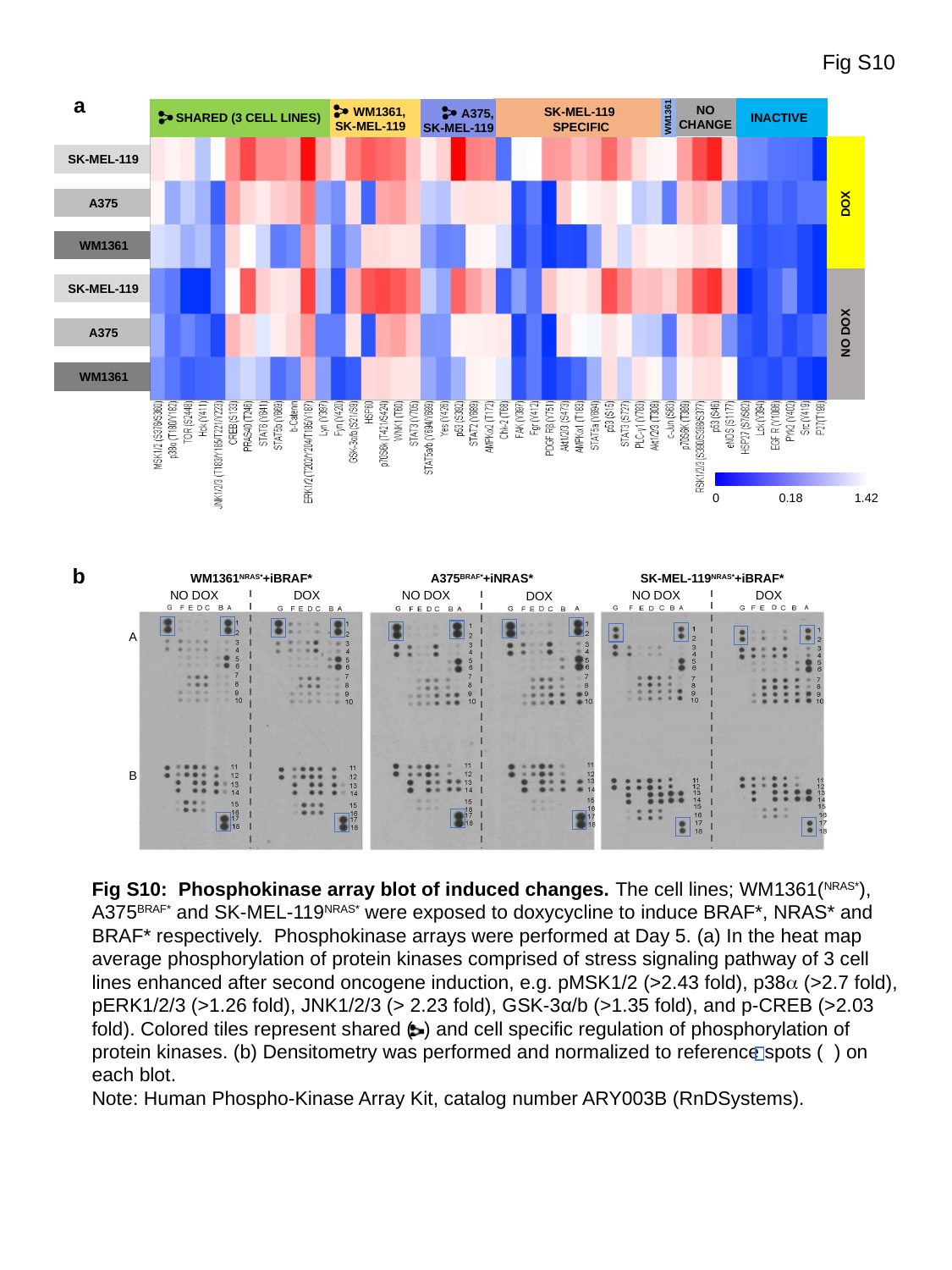

Fig S10
a
NO
CHANGE
WM1361,
SK-MEL-119
SK-MEL-119
SPECIFIC
A375,
SK-MEL-119
SHARED (3 CELL LINES)
INACTIVE
WM1361
SK-MEL-119
A375
DOX
WM1361
SK-MEL-119
A375
NO DOX
WM1361
0
0.18
1.42
b
WM1361NRAS*+iBRAF*
A375BRAF*+iNRAS*
SK-MEL-119NRAS*+iBRAF*
NO DOX
DOX
NO DOX
DOX
NO DOX
DOX
A
B
Fig S10: Phosphokinase array blot of induced changes. The cell lines; WM1361(NRAS*), A375BRAF* and SK-MEL-119NRAS* were exposed to doxycycline to induce BRAF*, NRAS* and BRAF* respectively. Phosphokinase arrays were performed at Day 5. (a) In the heat map average phosphorylation of protein kinases comprised of stress signaling pathway of 3 cell lines enhanced after second oncogene induction, e.g. pMSK1/2 (>2.43 fold), p38α (>2.7 fold), pERK1/2/3 (>1.26 fold), JNK1/2/3 (> 2.23 fold), GSK-3α/b (>1.35 fold), and p-CREB (>2.03 fold). Colored tiles represent shared ( ) and cell specific regulation of phosphorylation of protein kinases. (b) Densitometry was performed and normalized to reference spots ( ) on each blot.
Note: Human Phospho-Kinase Array Kit, catalog number ARY003B (RnDSystems).
