# Supplementary material for: Growth suppression by dual BRAF(V600E) and NRAS(Q61) oncogene expression is mediated by SPRY4 in melanoma
Source: Oncogene. 2019 Jan 16;38(18):3504–20. doi: 10.1038/s41388-018-0632-2 (PMC6756020; doi:10.1038/s41388-018-0632-2)
Supplement: Supplementary file 11 — supplementary figure 11 [file 41388_2018_632_MOESM11_ESM.pptx]

## Slide 1
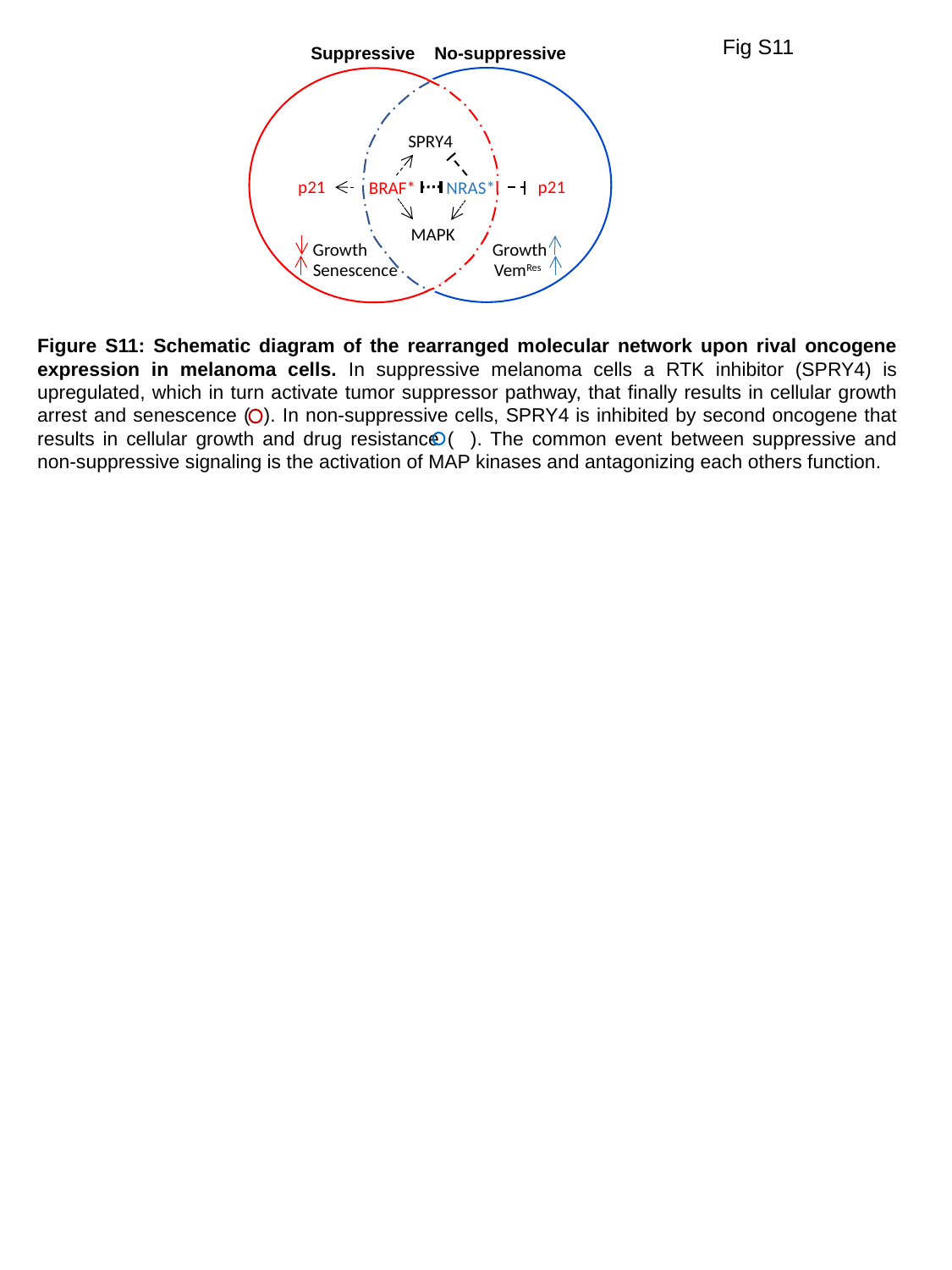

Fig S11
No-suppressive
Suppressive
SPRY4
p21
p21
BRAF*
NRAS*
MAPK
Growth
Growth
VemRes
Senescence
Figure S11: Schematic diagram of the rearranged molecular network upon rival oncogene expression in melanoma cells. In suppressive melanoma cells a RTK inhibitor (SPRY4) is upregulated, which in turn activate tumor suppressor pathway, that finally results in cellular growth arrest and senescence ( ). In non-suppressive cells, SPRY4 is inhibited by second oncogene that results in cellular growth and drug resistance ( ). The common event between suppressive and non-suppressive signaling is the activation of MAP kinases and antagonizing each others function.
