# Supplementary material for: Growth suppression by dual BRAF(V600E) and NRAS(Q61) oncogene expression is mediated by SPRY4 in melanoma
Source: Oncogene. 2019 Jan 16;38(18):3504–20. doi: 10.1038/s41388-018-0632-2 (PMC6756020; doi:10.1038/s41388-018-0632-2)
Supplement: Supplementary file 12 — Materials and Methods-SUPPLEMENTARY [file 41388_2018_632_MOESM12_ESM.docx]

**Supplementary**

**Materials and Methods**

**Molecular Cloning**

Following assays were conducted as previously described by our laboratory[^1^](#_ENREF_1)^,^ [^2^](#_ENREF_2). Lentiviral particles were used to deliver tetracycline inducible recombinant DNA constructs. Full-length BRAF^wt^ and BRAF^V600E^ were PCR amplified from pBABE-B-Raf, pBABE-B-Raf-V600E vector (Addgene) using primers (Forward:5'-ATATGGCCCCCGGGGACGCGTGCC*ATGGCGGCGCTGAGC*-3’ and Reverse: 5'-TCCCCTACCCGGTAGAATTCTCA*GTGGACAGGAAACGCAC*-3') with amplicon possessing the t1799a/v600e mutation. Similarly, pBABE-NRAS^WT^, pBABE-NRAS^Q61R^, and pBABE-NRAS^Q61K^ (Addgene) [Forward: 5'-ATATGGCCCCCGGGGACGCGTGCC*ATGACTGAGTACAAACTGGTG*-3', Reverse: 5'-TCCCCTACCCGGTAGAATTC*TTACATCACCACACATGGC*-3'] and human SPRY4 (Origene) (Forward: 5'-CCCGGACGCGTGCC*ATGCTCAGCCCCCTC*-3’ and Reverse: 5'-TACCCGGTAGAATTC*TCAGAAAGGCTTGTCGG*-3’) vectors were used to PCR amplify respective genes and cloned in lentiviral Tet-On pLVX-TRE3G-ZsGreen1 (Clontech) and constitutive CD516B-2 (System Biosciences, Palo Alto, CA) vectors. The ligation product was transformed into competent E. coli bacterial strain DH5α (NEB). The empty vector (pLVX-TRE3G-ZsGreen1) designated as “Tet-On vector” and over expressing recombinant constructs (pLVX-TRE3G-ZsGreen1-GENE), designated as “Tet-On GENE”, was used to generate stable cell lines.

**Lentiviral Production and Generation of Tet-inducible Stable Melanoma Cell lines**

293FT (ATCC, Manassas, VA) cells were plated in 60 mm dish at 3x10^6^ cells/plate. After 24 h, cells were transfected with 3.5 µg of target lentiviral construct, along with 17 µl Lenti-X HTX packaging Mix2 using 3.75 µl Xfect polymer and incubated overnight. The next day, fresh media was added, at 48 hours of post-transfection, supernatant was harvested and centrifuged briefly at 500 x g for 10 minutes to remove cellular debris as per the manufacturer’s instructions (Clontech). Virus-containing media was stored at -80 ºC until infection. A day prior of transduction, stable regulator (pLVX-EF1α-TET3G vector, G418 (0.5-2 mg/ml)) melanoma cells were plated at density 3×10^5^ cells per well with 2 ml DMEM growth medium (containing 10% tetracycline free FBS, designated as Tet-free medium) in 6-well plate. Next day, lentivirus supernatant of 0.1 to 0.2 ml of 5 ml (MOI:1.67) and 8 μg/ml of polybrene were added over cells containing fresh Tet-free medium in a total volume of 1 ml. Cells were incubated overnight at 37°C in humidified 5% CO_2_ incubator before replacing with 2 ml fresh Tet-free medium without antibiotics for expansion of the transductants. Cells were selected with puromycin at 2- 6 μM for another 15 days before further experiments. The stable clones were propagated, and protein expression levels were confirmed with Western blotting.

**Determination of the Transcript Copy number of BRAF and NRAS and its Comparison to Endogenous levels**

Total mRNA was collected from doxycycline induced and non-induced melanoma cell lines and 2 µg RNA was converted to cDNA (High Capacity RNA-to-cDNA Kit). The Wagatsuma method[^6^](#_ENREF_6) was used to determine the exact transcript copy numbers of BRAF and NRAS, both of which was compared with two standard curves plotted with the threshold cycles (Cq values) from recombinant plasmid (rDNA) and sample cDNA diluted 1/10 five serial dilutions. The standards of sample cDNA are from Tet-On BRAF^V600E^ SK-MEL-119^NRAS*^ designated as “SK-MEL-119^NRAS*^+iBRAF*”, or Tet-On NRAS^Q61R^ GMEL^BRAF*^ designated as “GMEL^BRAF*^+iNRAS*” cell lines induced with doxycycline 100 ng/ml for 3 days. We first obtained the standard curves of BRAF and NRAS using recombinant plasmids (pcDNA-NRAS^Q61R^ or pcDNA-BRAF^V600E^) solutions. The Copy numbers of recombinant plasmid (rDNA) was within the range of 10^1^ to 10^5^ copies per reaction, and the standard curve from SK-MEL-119^NRAS*^+iBRAF* or from GMEL^BRAF*^+iNRAS* cDNA was parallel (arbitrary units) to their corresponding recombinant plasmid standard curve (Fig. S1a, b). The standard curve slopes/E-amp (amplification efficiency) for the two target genes were (BRAF: -3.2377/2.036 for the recombinant plasmid (rDNA) standard and -3.3810/1.976 for the sample cDNA solutions and NRAS: -2.9053/2.209 for the recombinant plasmid (rDNA) standard and -3.4705/1.941 for the sample cDNA solutions). These results confirm that samples amplify target BRAF and NRAS nearly at same efficiency as rDNA plasmid solutions, respectively.

To get transcript copy numbers, we took the efficiencies of both the standards into an account and calculated the copy number of mRNA[^4^](#_ENREF_4)^,^ [^6^](#_ENREF_6). Next, we determined the transcript copy number of BRAF and NRAS mRNA in a broader panel of cell lines and doxycycline induced or non-induced SK-MEL-119^NRAS*^+iBRAF* and GMEL^BRAF*^+iNRAS* melanoma cells (Fig. S1c, d). The mRNA copies per micro liter of the sample solution at 0 to 1000 ng per ml doxycycline at 3^rd^ day was determined to range from 1.54x10^6^ to 99.97x10^6^ for BRAF mRNA in SK-MEL-119^NRAS*^+iBRAF* and 81.04.25x10^6^ to 4847.11x10^6^ for NRAS mRNA in GMEL^BRAF*^+iNRAS* and RNA expression found to be well sustained in all the melanoma lines. Hence, by calculating the average value of triplicate samples at 50 to 100ng per ml doxycycline in respective cell lines, the transcript copy number after induction for BRAF was from 27.66x10^6^ to 42.48x10^6^ in SK-MEL-119^NRAS*^+iBRAF* which is in nearly parallel to A373-C6 and K2 cells and for NRAS was from 1373.25x10^6^ to 2092.42x10^6^ which is in almost parallel to A373-C6 and SK-MEL-28 cell lines. Thus, the mRNA copy number obtained with 50 to 100ng per ml doxycycline were close to naïve cell lines. Therefore, the induction was in physiological range and comparable to endogenous level of many melanoma cell line (Fig. S1c, d).

**Western Blot Analysis and Phospho-kinase Profile**

Cells with indicated conditions (Doxycycline: +Dox, 50-100ng/ml or No-Doxycycline: -Dox) were screen harvested, washed with 1x phosphate-buffered saline and lysed in a RIPA buffer (Boston Bioproducts, Ashland, MA) supplemented with halt protease inhibitor cocktail (Thermo Scientific, Rockford, IL). The protein concentrations of the lysates were measured by BCA protein assay kit (BioRad, Hercules, CA). The lysates (10–20 μg protein) were separated on 4–20% SDS polyacrylamide mini-gels (Bio-Rad, Hercules, CA) and transferred to PVDF or nitrocellulose membrane followed by Western Blot Analysis as described previously (Kumar et al., 2015). The antibodies used were as follows: mouse monoclonal anti-BRAF^V600E^ (1:4000, Spring Bioscience, Pleasanton, CA), NRAS^Q61R^ (1:2000, Abnova, Atlanta, GA), anti-Sprouty4, NRAS^WT^, BRAF^WT^ (1:1000, Santa Cruz Biotechnology, Dallas, Texas), and mouse monoclonal anti-GAPDH (1:40,000, Abcam, Cambridge, MA) for 2 hours, and Horseradish peroxidase– conjugated secondary antibody (1:4,000) for 1hour. Antigen–antibody complexes were detected by ECL enhanced chemiluminescence solution (Bio-Rad, Hercules, CA). The signal intensity was quantified using ImageJ analysis software[^5^](#_ENREF_5). To clearly demonstrate the difference, the relative gray-scale value of target protein vs GAPDH of the control group was set as 1. Results shown are representative of three independent experiments. Phospho-kinase screening was performed using a phospho-kinase array (R&D systems, Inc., Minneapolis, MN, ARY003B) as per the manufacturer’s instructions. Briefly, as discussed above, whole cell lysates of 1x10^7^ cells/ml were subjected for phosphokinase array and for each cell line, the densitometric values were normalized to reference spots on each immunoblots and compared in fold change with uninduced sample (Fig S10).

**Apoptosis and Cell cycle**

Cells, washed with PBS, incubated with Annexin-V Alexa Fluor 647 and DAPI for 15 minutes at room temperature in the dark as per the manufacturer’s protocol (Life Technologies). The percentage of Annexin-V positive cells was determined by flow cytometry BD FACSAria, (BD Biosciences-US) and results were analyzed using FlowJo, version 7.6.5 software (Ashland, OR).

Cell cycle analyses were performed to evaluate the distribution of cells in various cell cycle phases (subG1, G1, S, and G2/M) by measuring the DNA content of nuclei labeled with propidium iodide (PI) (Life Technologies). Cells were fixed with ice-cold 70% (v/v) ethanol drop wise while gently vortexing and kept at -20 ºC overnight prior to PI staining and flow cytometric analysis. Cells were centrifuged and washed twice with cold 1xPBS and added 0.5 ml of propidium iodide staining solution to cell pellet and mix well (100 µg/ml of propidium iodide and 100 µg/ml of RNase A in 0.1%Triton X-100) in 1xPBS (Sigma Aldrich, St. Louis, MO) and incubated at room temperature in dark for 30 minutes (Kumar et al., 2015). Samples were analyzed by flow cytometry (BD FACS Calibur flow cytometer, BD Biosciences-US, Sparks Glencoe, MD). FlowJo, version 7.6.5 software (Ashland, OR) was used to calculate the percentages of cells in various cell cycle phases. As stated before, the cell cycle and apoptosis assays were performed in parallel and in triplicate for each condition.

**Senescence Assay**

Cell senescence was measured by detection of SA-β-gal activity using the Senescence Detection Kit (K#320-250, BioVision, Milpitas, CA) according to manufacturer’s instruction. To prepare oncogene-induced secondary senescent melanoma cells for these assays, Tet-On BRAF^V600E^ (SK-MEL-119^NRAS*^+iBRAF*, WM1361^NRAS*^+iBRAF*, and SK-MEL-63^NRAS*^+iBRAF*) and Tet-On NRAS^Q61R^ (GMEL^BRAF*^+iNRAS*, and A375^BRAF*^+iNRAS*) viable melanoma cells were induced with or without doxycycline (50-100ng/ml) for 3 days. The cells were then subjected to senescence-associated expression of β-galactosidase (SA-β-gal assay) activity as per manufacturer’s instruction (BioVision, Milpitas, CA).

**Q-PCR Analysis**

Cells were seeded in 10 cm plates at a density of 1.0x10^6^cells/well and allowed to adhere overnight in Tet-free growth medium. Next day, medium was replaced with or without doxycycline 50-100ng/ml for a defined time of 4-5 days before total RNA was isolated using RNeasy isolation kit (Qiagen, Germany) according to manufacturer’s instructions. The first strand cDNA was reversely transcribed from 2 micrograms of RNA using high capacity RNA to cDNA kit according to manufacturer’s instructions (Applied Biosystems, Foster City, CA, USA). Microarray data was verified by qPCR using the gene-specific primers [Invitrogen; SPRY4(Hs01935412_s1), NRAS (Hs 00180035-m1), BRAF (Hs 00269944-m1), and: *Hu-GUSB-FAM*-4333767F], IDT: hMITF-M[^1^](#_ENREF_1) and TaqMan Master Mix (Roche Diagnostics Cor. Indianapolis, IN, USA). The relative expression of target genes versus a reference gene, *Hu-GUSB-FAM*, was calculated using Ct values/or a built-in mathematical model (Roche Molecular Diagnostics, Branchburg, NJ) which included an efficiency correction for real time PCR.

**Microarray Preparation and Analysis**

Cells were seeded 24 hours before treatment with or without doxycycline 50-100ng/ml for a defined time of 4-5 days. RNA was extracted from 2x10^6^ cells from each condition (biologic duplicates) using the RNeasy Mini Kit (Qiagen). The RNA specimens were submitted to the MIT Core Facility and profiled with Affymetrix Primeview GeneChip arrays as per standard operating procedures of the Core Facility. Probe set intensity values were converted into log_2_ space after adding a pseudo-count of 1. Expressed genes were those with log_2_(expression) > log_2_(100 units), which is approximately log_2_ ≈ 6.64. Only transcript probes which were expressed in at least 3 or more samples were used for the comparative analysis. The effect of BRAF* or NRAS* was quantified as log_2_-fold differences using the formula:

Oncogene effectlog_2-fold_ = [Oncogene(+Dox) log_2-expression_ ─ Oncogene(-Dox) log_2-expression_] ─ [Vector(+Dox) log_2-expression_ ─ Vector(-Dox) log_2-expression_]. The entire expression set in log_2_ expression levels is shown in Table S1.

**Functional Enrichment Analysis Using DAVID**

Probes that exhibited >2-fold change (+1.0 or -1.0 log_2_-fold) for each line (Tables S2-S4) and for probes that were shared in SK-MEL-119^NRAS*^+iBRAF* and GMEL^BRAF*^+iNRAS* but NOT A375^BRAF*^+iNRAS* (Table S5) were subjected to DAVID V6.8 (<https://david.ncifcrf.gov/> ) functional clustering annotation under the default mode. Per DAVID’s website for functional clustering, “The geometric mean (in -log scale) of member's p-values in a corresponding annotation cluster, is used to rank their biological significance. Thus, the top ranked annotation groups most likely have consistent lower p-values for their annotation members.” Annotation clustering Enrichment Scores are presented in Tables S2-S5. The annotation cluster is designated by the first GO term within the cluster, which by convention, has the lowest p-value in the cluster.

**Immunohistochemistry, H&E and TUNEL**

Immunohistochemistry, phosphate-buffered formalin fixed tumor tissues were paraffin-embedded and 5µM thick sections were collected onto poly-L-lysine–coated slides. The assays were performed as described previously[^3^](#_ENREF_3). Briefly, the sections were subjected for IHC, H&E and TUNEL. Two archival tumor sections per slide were deparaffinized at 60°C for 1h followed by a few CitriSolv dips and gradually rehydrated through graded ethanol. 1) IHC, Antigen retrieval (1xDAKO, Cat# S1699) was performed at 98°C water bath for 30 minutes, permeabilization in 0.1% TritonX-100 TBS at 25°C for 15 minutes. Endogenous AP and peroxidase blocking in 3% H_2_O_2_ solution for 15min at 25°C followed by protein blocking serum (10% Goat serum, 3% BSA, 0.1% Tween-20 in TBS Sigma-Aldrich, Natick, MA) for 30 minutes. After this, tissue sections were Incubated for 1 hour, with primary antibodies such as anti-Ki67 for proliferation, anti-sprouty4 for over expression, β-galactosidase for senescence, followed by a secondary alkaline phosphatase antibody, either MACH2 mouse AP-polymer detection, (BioCare Medical, Cat# MALP521-G) or MACH 2 rabbit AP-polymer detection, (BioCare Medical, Cat# RALP525-G) for 30 minutes. The slides were developed with Vulcan Fast Red Chromogen kit2 (BioCare Medical, Cat# FR805 H) and counter stained with Mossberg hematoxylin (Vector H-3401). The colorimetric signals were detected with digital slide scanner NanoZoomer-2.0HT and analyzed by NDP.view2 software (Hamamatsu Photonics K.K., Hamamatsu, Japan). 2) H&E, after rehydration hematoxylin and eosin staining was carried out using the Mossberg labs protocol. 3)TUNEL staining was performed using Dead End Fluorometric TUNEL kit (Promega) according to manufactures instruction [^2^](#_ENREF_2)

**Cell Line Authentication**

All cell lines were stratified to 3 levels of confidence after STR genotyping and *MITF* expression analysis. Those lines that matched the STR genotyping database were considered “CONFIRMED” as the designated line. Cell lines with no STR hits but had mutational data at melanoma driver loci (e.g. BRAF, NRAS, PTEN, etc.) were manually compared to public datasets such as COSMIC or individually referenced papers. If the mutational profile of the cell line matched independent public data (i.e. results not from our laboratory), these lines were labeled “CONSISTENT” with the designated line. Lastly, cell lines that were either newly developed or lacked STR information and public domain information were experimentally assayed for *MITF* levels by RNAseq. Those that showed *MITF* expression were considered “COMPATIBLE” with melanoma. The following represents the level of confirmation for cell lines in this study: A375^BRAF*^ (CONFIRMED), GMEL^BRAF*^ (CONFIRMED), WM1361^NRAS*^ (CONFIRMED), SK-MEL-63^NRAS*^ (CONSISTENT) and MGH-CH-1^BRAF*^ (COMPATIBLE). The cell lines were thawed and collected between 3^rd^ or 4^th^ passages for original naive and stably expressing transductant cells. After experiment, the cell lines were either used up or discarded at 15^th^ to 25^th^ day. All used cell lines were mycoplasma tested as instructed by the company (Invitrogen).

# References

1 Ji Z, Erin Chen Y, Kumar R, Taylor M, Jenny Njauw CN, Miao B *et al* (2015). MITF Modulates Therapeutic Resistance through EGFR Signaling. *J Invest Dermatol* **135:** 1863-1872.

2 Kumar R, Taylor M, Miao B, Ji Z, Njauw JC, Jonsson G *et al* (2015). BAP1 has a survival role in cutaneous melanoma. *J Invest Dermatol* **135:** 1089-1097.

3 Miao B, Ji Z, Tan L, Taylor M, Zhang J, Choi HG *et al* (2015). EPHA2 is a mediator of vemurafenib resistance and a novel therapeutic target in melanoma. *Cancer Discov* **5:** 274-287.

4 Opriessnig T, Yu S, Gallup JM, Evans RB, Fenaux M, Pallares F *et al* (2003). Effect of vaccination with selective bacterins on conventional pigs infected with type 2 porcine circovirus. *Vet Pathol* **40:** 521-529.

5 Schneider CA, Rasband WS, Eliceiri KW (2012). NIH Image to ImageJ: 25 years of image analysis. *Nature methods* **9:** 671-675.

6 Wagatsuma A, Sadamoto H, Kitahashi T, Lukowiak K, Urano A, Ito E (2005). Determination of the exact copy numbers of particular mRNAs in a single cell by quantitative real-time RT-PCR. *The Journal of experimental biology* **208:** 2389-2398.
